# Supplementary material for: Intake of dietary fats and fatty acids and the incidence of type 2 diabetes: A systematic review and dose-response meta-analysis of prospective observational studies
Source: PLoS Med. 2020 Dec 2;17(12):e1003347. doi: 10.1371/journal.pmed.1003347 (PMC7710077; doi:10.1371/journal.pmed.1003347)
Supplement: S2 Fig — (DOCX) [file pmed.1003347.s003.docx]

**S2 Fig**: Linear dose-response meta-analyses on the associations between A) total fat, B) animal fat and C) vegetable fat and incidence of type 2 diabetes

| A) |   , tau^2^=0.003  Goodness-of-fit chi^2^=32.02 |
| --- | --- |
| B) |   , tau^2^=0.000  Goodness-of-fit chi^2^=2.37 |
| C) |   Goodness-of-fit chi^2^=37.1  , tau^2^=0.018 |
