# Supplementary material for: Intake of dietary fats and fatty acids and the incidence of type 2 diabetes: A systematic review and dose-response meta-analysis of prospective observational studies
Source: PLoS Med. 2020 Dec 2;17(12):e1003347. doi: 10.1371/journal.pmed.1003347 (PMC7710077; doi:10.1371/journal.pmed.1003347)
Supplement: S3 Fig — (DOCX) [file pmed.1003347.s004.docx]

**S3 Fig:** Linear dose-response meta-analyses on the associations between A) saturated fatty acids, B) monounsaturated fatty acids, C) polyunsaturated fatty acids, D) omega-6 fatty acids, E) linoleic acid, F) omega-3 fatty acids, G) long-chain omega-3 fatty acids (EPA&DHA), H) eicosapentaenoic acid, I) docosahexaenoic acid, J) alpha linolenic acid, K) omega-6:omega-3-ratio and L) trans-fatty acids and incidence of type 2 diabetes

| A) |   , tau^2^=0.002  Goodness-of-fit chi^2^=15.2 |
| --- | --- |
| B) |   , tau^2^=0.000  Goodness-of-fit chi^2^=6.61 |
|  |  |
| C) |   Goodness-of-fit chi^2^=29.6  , tau^2^=0.033 |
| D) |   Goodness-of-fit chi^2^=23.6  , tau^2^=0.000 |
| E) |   Goodness-of-fit chi^2^=14.2  , tau^2^=0.000 |
| F) |   Goodness-of-fit chi^2^=30.2  , tau^2^=0.002 |
|  |  |
| G) |   Goodness-of-fit chi^2^=70.9  , tau^2^=0.007 |
|  |  |
| H) |   Goodness-of-fit chi^2^=15.7  , tau^2^=0.040 |
|  |  |
| I) |   Goodness-of-fit chi^2^=27.3  , tau^2^=0.029 |
|  |  |
| J) |   Goodness-of-fit chi^2^=29.0  , tau^2^=0.002 |
|  |  |
| K) |   Goodness-of-fit chi^2^=0.0  , tau^2^=0.000 |
| L) |   Goodness-of-fit chi^2^=17,9  , tau^2^=0.003 |
