# Supplementary material for: Intake of dietary fats and fatty acids and the incidence of type 2 diabetes: A systematic review and dose-response meta-analysis of prospective observational studies
Source: PLoS Med. 2020 Dec 2;17(12):e1003347. doi: 10.1371/journal.pmed.1003347 (PMC7710077; doi:10.1371/journal.pmed.1003347)
Supplement: S4 Fig — (DOCX) [file pmed.1003347.s005.docx]

**S4 Fig**: Sensitivity analyses for A) total fat, B) animal fat and C) vegetable fat

| A) |  | 1.01 (0.95, 1.06)  1.00 (0.95, 1.06)  1.00 (0.95, 1.06)  0.99 (0.95, 1.03)  0.99 (0.95, 1.05)  1.01 (0.95, 1.07)  0.98 (0.94, 1.03)  1.01 (0.98, 1.05) |
| --- | --- | --- |
| B) |  | 1.03 (1.00, 1.06)  1.03 (1.00, 1.07)  1.02 (0.99, 1.06)  1.04 (1.00, 1.08)  1.02 (0.99, 1.06) |
| C) |  | 0.88 (0.79, 0.98)  0.93 (0.78, 1.12)  0.93 (0.80, 1.08)  0.98 (0.89, 1.08)  0.93 (0.79, 1.09) |
