# Supplementary material for: Intake of dietary fats and fatty acids and the incidence of type 2 diabetes: A systematic review and dose-response meta-analysis of prospective observational studies
Source: PLoS Med. 2020 Dec 2;17(12):e1003347. doi: 10.1371/journal.pmed.1003347 (PMC7710077; doi:10.1371/journal.pmed.1003347)
Supplement: S5 Fig — (DOCX) [file pmed.1003347.s006.docx]

**S5 Fig**: Sensitivity analyses for A) saturated fatty acids, B) monounsaturated fatty acids, C) polyunsaturated fatty acids, D) omega-6 fatty acids, E) linoleic acid, F) omega-3 fatty acids, G) long-chain omega-3 fatty acids (EPA&DHA), H) eicosapentaenoic acid, I) docosahexaenoic acid, J) alpha linolenic acid and K) trans-fatty acids

| A) |  | 0.98 (0.93, 1.03)  0.96 (0.91, 1.01)  0.98 (0.91, 1.05)  0.97 (0.91, 1.02)  0.97 (0.93, 1.02)  0.96 (0.92, 1.00)  0.97 (0.91, 1.02)  0.97 (0.91, 1.03)  0.96 (0.91, 1.02)  0.97 (0.92, 1.03)  0.97 (0.91, 1.03) |
| --- | --- | --- |
| B) |  | 1.02 (0.97, 1.07)  1.03 (0.98, 1.08)  1.04 (0.98, 1.09)  1.03 (0.98, 1.08)  1.03 (0.99, 1.08)  1.03 (0.98, 1.08)  1.05 (1.00, 1.10)  1.03 (0.99, 1.08)  1.03 (0.98, 1.08)  1.03 (0.98, 1.07) |
| C) |  | 1.00 (0.86, 1.18)  1.03 (0.86, 1.23)  1.04 (0.85, 1.26)  1.00 (0.86, 1.17)  1.01 (0.86, 1.19)  1.07 (0.90, 1.26)  1.03 (0.87, 1.22)  1.09 (0.98, 1.21) |
| D) |  | 0.99 (0.98, 1.00)  0.99 (0.98, 1.01)  0.99 (0.98, 1.01)  0.99 (0.98, 1.00)  0.99 (0.98, 1.01)  1.00 (0.98, 1.01)  0.99 (0.98, 1.01)  1.00 (0.99, 1.01) |
| E) |  | 0.99 (0.98, 1.01)  0.99 (0.98, 1.00)  0.99 (0.98, 1.00)  1.00 (0.98, 1.01)  0.99 (0.98, 1.01)  1.00 (0.99, 1.01) |
| F) |  | 1.07 (0.88, 1.29)  1.21 (1.00, 1.47)  1.04 (0.88, 1.23)  1.16 (0.87, 1.54)  1.13 (0.89, 1.42) |
| G) |  | 1.06 (1.00, 1.11)  1.07 (1.01, 1.12)  1.04 (1.00, 1.09)  1.06 (1.01, 1.12)  1.06 (1.00, 1.12)  1.06 (1.01, 1.12)  1.05 (0.99, 1.11)  1.05 (0.99, 1.10)  1.05 (1.00, 1.12)  1.05 (1.00, 1.11)  1.05 (1.00, 1.11)  1.06 (1.01, 1.12)  1.07 (1.02, 1.13)  1.06 (1.01, 1.12)  1.06 (1.01, 1.11)  1.06 (1.00, 1.11) |
| H) |  | 1.05 (0.72, 1.53)  0.98 (0.82, 1.19)  1.20 (0.97, 1.50)  1.11 (0.87, 1.41) |
| I) |  | 1.14 (0.84, 1.57)  1.04 (0.98, 1.11)  1.15 (0.84, 1.56)  1.16 (0.95, 1.42) |
| J) |  | 1.00 (0.97, 1.03)  1.02 (0.99, 1.06)  1.01 (0.98, 1.05)  1.01 (0.98, 1.05)  1.01 (0.98, 1.05)  1.02 (0.97, 1.07)  1.01 (0.98, 1.05)  1.01 (0.97, 1.05)  1.01 (0.97, 1.05)  1.01 (0.97, 1.04)  1.02 (0.98, 1.05) |
| K) |  | 1.00 (0.94, 1.07)  1.00 (0.95, 1.07)  1.02 (0.97, 1.08)  0.97 (0.93, 1.01)  1.00 (0.94, 1.08)  0.99 (0.93, 1.05)  1.01 (0.94, 1.09) |
