# Supplementary material for: Intake of dietary fats and fatty acids and the incidence of type 2 diabetes: A systematic review and dose-response meta-analysis of prospective observational studies
Source: PLoS Med. 2020 Dec 2;17(12):e1003347. doi: 10.1371/journal.pmed.1003347 (PMC7710077; doi:10.1371/journal.pmed.1003347)
Supplement: S6 Fig — Nonlinear dose–response meta-analyses for the association between long-chain omega-3 fatty acids and incidence of type 2 diabetes stratified by geographic region. (DOCX) [file pmed.1003347.s007.docx]

**S6 Fig**: Non-linear dose-response meta-analyses for the association between long-chain omega-3 fatty acids and incidence of T2D stratified by geographic region for A) USA, B) Europe, C) Asia.

| A) |  |
| --- | --- |
| B) |  |
| C) |  |
