# Supplementary material for: Intake of dietary fats and fatty acids and the incidence of type 2 diabetes: A systematic review and dose-response meta-analysis of prospective observational studies
Source: PLoS Med. 2020 Dec 2;17(12):e1003347. doi: 10.1371/journal.pmed.1003347 (PMC7710077; doi:10.1371/journal.pmed.1003347)
Supplement: S7 Fig — (DOCX) [file pmed.1003347.s008.docx]

**S7 Fig**: Funnel plots for A) saturated fatty acids (n=11), B) monounsaturated fatty acids (n=10), C) long-chain omega-3 fatty acids (n=16) and D) alpha-linolenic acid (n=11)

| A) |  |
| --- | --- |
| B) |  |
| C) |  |
| D) |  |
