# Supplementary material for: Intake of dietary fats and fatty acids and the incidence of type 2 diabetes: A systematic review and dose-response meta-analysis of prospective observational studies
Source: PLoS Med. 2020 Dec 2;17(12):e1003347. doi: 10.1371/journal.pmed.1003347 (PMC7710077; doi:10.1371/journal.pmed.1003347)
Supplement: S1 Table — (DOCX) [file pmed.1003347.s009.docx]

**S1 Table**: Description and decision criteria for each domain in ROBINS-I

| **Domain** | **Explanation** | **Judgements** |
| --- | --- | --- |
| **Bias due to confounding** | - Is there potential for confounding of the effect of exposure in this study? - Did the authors use a multivariable-adjusted analysis method that controlled at least for age, sex, smoking, education/socioeconomic status and total energy intake? - Did the authors avoid adjusting for post-exposure variables?   Notes: Confounding is expected in all observational studies, low risk of bias was not assigned to any study. Time-varying confounding was expected to be unlikely and is not expected to cause risk of bias in the present study. | Low risk of bias: No bias expected due to confounding, including time-varying confounding.  Moderate risk of bias: Confounding is expected: age, sex, smoking, education/socioeconomic status and total energy intake have been appropriately controlled for in a multivariable-adjusted analysis  *or* confounding is expected: age, sex, smoking, and total energy intake have been appropriately controlled for in a multivariable-adjusted analysis *and* education/socioeconomic status is not expected to vary substantially within the cohort (e.g. NHS, HPFS)  *or* the authors statistically investigated whether the confounding domains have an effect on the risk estimate and excluded the confounder from the multivariable model if there was no effect on the overall effect estimate.  Serious risk of bias: At least one known important domain was not measured or appropriately controlled for  No information: No information on which confounder have been controlled for. |
| **Bias due to selection of participants** | - Was selection of participants into the study based on participants characteristics observed after start of the study/exposure assessment? - Do start of follow-up and start of exposure coincide for most participants? Were methods used that are likely to correct for the presence of selection biases?   Notes: In observational studies, it is unlikely that post-exposure variables influenced selection of participants into the study. Exclusion of participants may be mostly based on missing data, which will be considered in the domain referring to missings (see below). The start of follow-up is considered to coincide with the baseline exposure assessment. However, participants are already exposed at start of the study which might have influenced outcome measured that occurred shortly after start of the study. | Low risk of bias: All participants who would have been eligible for the target study were included in the study; *and* the authors conducted a sensitivity analysis excluding T2D cases which occurred <2 years after start of the study and the results did not change.  Moderate risk of bias: Selection into the study may have been related to exposure and outcome (e.g. inclusion of postmenopausal women only); *and* the authors used appropriate methods to correct for the selection bias;  *or* the authors conducted no sensitivity analysis excluding T2D cases which occurred <2 years after start.  Serious risk of bias: Selection into the study was related to exposure and outcome (e.g. only participants with prediabetes were included in the analysis); *and* this could not be corrected for in the analyses;  *or* start of follow up and start of exposure do not coincide *and* the rate ratio is not constant over time.  No information: No information is reported about selection of participants into the study. |
| **Bias due to exposure assessment** | - Were exposure groups clearly defined and adequately assessed? - Was the information used to define the exposure groups based on reasonable a priori data?   Note: The start of follow-up is considered to coincide with the baseline exposure assessment. Any dietary assessment method involves measurement error (Imamura 2015, *BMJ*), no study was assigned low risk of bias. | Low risk of bias: Exposure status was well defined (comprehensibly derived categories); *and* no measurement error is expected in its assessment.  Moderate risk of bias: Exposure status is well defined (comprehensibly derived categories); *and* exposure was measured using a validated tool (e.g. a validated FFQ).  Serious risk of bias: Exposure status is not well defined; *and* exposure was measured using not validated tools.  No information: No definition of exposure or no explanation of the source of information about exposure status is reported. |
| **Bias due to misclassification during follow-up** | - Were there deviations from the exposure beyond what would be expected in usual practice? - Were these deviations unbalanced between groups and likely to have affected the outcome?   Notes: Repeated measurements of the exposure are mostly not available in observational studies. It is not expected that there are high changes in diet in healthy participants. Changes in diet may be similar between studies and may also be similar between groups (differential misclassification is not expected). Recent studies have shown that diet is constant or change only slightly over time (Feldman 2017 IJBNPA, VanWormer 2017 Preventive Medicine Reports). Thus, if repeated measures are not available, moderate risk of bias could be assigned to a study. | Low risk of bias: Repeated measurements of the exposure status during follow-up are available. No or only slightly changes in fat intake were observed and the changes were considered in the analysis.  Moderate risk of bias: Repeated measurements of the exposure are not available, but high changes are not expected during follow-up (compare notes)  or repeated measurements of the exposure status during follow-up are available and some changes in lifestyle factors were observed. The analysis was appropriate to estimate the effect of changes in lifestyle factors, allowing for deviations that were likely to impact on the outcome;  Serious risk of bias: Exposure status is measured during follow-up and high changes in lifestyle factors have been observed, and the analysis was not appropriate to estimate the effect of changes in lifestyle factors, allowing for deviations that were likely to impact on the outcome.  No information: No information on deviations from the exposure is reported. |
| **Bias due to missing data** | - Were there missing outcome data? - Were participants excluded due to missing data on exposure status? - Were participants excluded due to missing data on other variables needed for analysis?   Notes: Missing data on exposure variables and other variables are expected to be missing at random and not related to exposure or outcome that have been assessed during follow-up. | Low risk of bias: Little loss-to-follow-up (<20%) and data on exposure and other variables were reasonably complete (<10% missing data) and was unlikely to introduce bias;  *or* the analysis addressed missing data and is likely to have removed any risk of bias.  Moderate risk of bias: There is a proportion of missing data in the original cohort or a high proportion of loss-to-follow-up; *and* the analysis is unlikely to have removed the risk of bias arising from the missing data (e.g. using logistic regression).  Serious risk of bias: High proportions (>50%) of missing data; *and* the analysis is unlikely to have removed the risk of bias arising from the missing data;  *or* missing data were addressed inappropriately in the analysis;  *or* the nature of the missing data means that the risk of bias cannot be removed through appropriate analysis.  No information: No information is reported about missing data or the potential for data to be missing. |
| **Bias due to measurement of the outcome** | - Could the outcome measure have been influenced by knowledge of the exposure status? - Were the methods of outcome assessment comparable across exposure groups? - Were any systematic error in measurement of the outcome related to exposure status?   Notes: In observational studies, it is not expected that outcome assessors were aware of exposure status of the participants. | Low risk of bias: The methods of outcome assessment were comparable across exposure groups; *and* the outcome measure was unlikely to be influenced by knowledge of the exposure status of study participants; *and* any error in measuring the outcome is unrelated to exposure status (i.e. objective measures such as confirmed medical records, record linkage).  Moderate risk of bias: The methods of outcome assessment were comparable across exposure groups; *and* any error in measuring the outcome may be minimally related to exposure status *or* if the outcome measure was not reliable measured (i.e. confirmed records are not available for the whole study population).  Serious risk of bias: The methods of outcome assessment were not comparable across exposure groups;  *or* the outcome measure was subjective (i.e. self-report of type 2 diabetes by study participants); *and* error in measuring the outcome was related to exposure status.  No information: No information is reported about the methods of outcome assessment. |
| **Bias due to selective reporting of the results** | - Is the reported effect estimate likely to be selected from multiple analyses of exposure-outcome relationship? - Is the reported effect estimate likely to be selected from different subgroups?   Notes: In observational studies, it is unusual to publish an a priori analysis plan or protocol. Multiple outcome measurements for the definition of type 2 diabetes are not expected. | Low risk of bias: There is a clear description of all analysis and the analyses are consistent and all reported results correspond to all intended outcomes, analyses and sub-cohorts.  Moderate risk of bias: The analyses are clearly defined; *and* there is indication of selection of the reported analysis from among multiple analyses; *and* there is indication of selection of the cohort or subgroups for analysis and reporting on basis of the results (e.g. estimates not shown for all analyses).  Serious risk of bias: There is a high risk of selective reporting from among multiple analyses; *or* the cohort or subgroup is selected from a larger study for analysis and appears to be reported based on the results.  No information: There is too little information to make a judgement. |
| **Overall judgement** | Low risk of bias | The study is judged to be at low risk of bias for all domains. |
|  | Moderate risk of bias | The study is judged to be at low or moderate risk of bias for all domains. |
|  | Serious risk of bias | The study is judged to be at serious risk of bias in at least one domain, but not at critical risk in any domain. |
