# Supplementary material for: Intake of dietary fats and fatty acids and the incidence of type 2 diabetes: A systematic review and dose-response meta-analysis of prospective observational studies
Source: PLoS Med. 2020 Dec 2;17(12):e1003347. doi: 10.1371/journal.pmed.1003347 (PMC7710077; doi:10.1371/journal.pmed.1003347)
Supplement: S2 Table — (DOCX) [file pmed.1003347.s010.docx]

**S2 Table:** List of excluded studies

| Not relevant outcome SRef [1-18] |
| --- |
| Not relevant exposure SRef [19-122] |
| Not relevant data SRef [123-127] |
| No risk estimates and/or 95% confidence intervals reported SRef [128, 129] |
| Case-control study SRef [130-132] |
| Cross-sectional study SRef [133] |
| Review / meta-analysis SRef [134-137] |
| Conference abstract SRef [138-153] |
| Letter / comment / report SRef [154] |
| Duplicate publication of the same study SRef [155-160] |
| Only data for high vs low analysis available SRef [161-166] |

**References**

1. Ascherio A, Rimm EB, Giovannucci EL, Spiegelman D, Stampfer M, Willett WC. Dietary fat and risk of coronary heart disease in men: cohort follow up study in the United States. BMJ. 1996;313(7049):84-90.

2. Jarvinen R, Knekt P, Rissanen H, Reunanen A. Intake of fish and long-chain n-3 fatty acids and the risk of coronary heart mortality in men and women. BrJ Nutr. 2006;95(4):824-9.

3. Li Y, Hruby A, Bernstein AM, Ley SH, Wang DD, Chiuve SE et al. Saturated Fats Compared With Unsaturated Fats and Sources of Carbohydrates in Relation to Risk of Coronary Heart Disease: A Prospective Cohort Study. J AmCollCardiol. 2015;66(14):1538-48.

4. Um YJ, Oh SW, Lee CM, Kwon HT, Joh HK, Kim YJ et al. Dietary Fat Intake and the Risk of Metabolic Syndrome in Korean Adults. Korean J FamMed. 2015;36(5):245-52.

5. Kim YS, Xun P, Iribarren C, Van HL, Steffen L, Daviglus ML et al. Intake of fish and long-chain omega-3 polyunsaturated fatty acids and incidence of metabolic syndrome among American young adults: a 25-year follow-up study. EurJ Nutr. 2016;55(4):1707-16.

6. Dehghan M, Mente A, Zhang X, Swaminathan S, Li W, Mohan V et al. Associations of fats and carbohydrate intake with cardiovascular disease and mortality in 18 countries from five continents (PURE): a prospective cohort study. Lancet. 2017;390(10107):2050-62.

7. Charles MA, Eschwege E, Thibult N, Claude JR, Warnet JM, Rosselin GE et al. The role of non-esterified fatty acids in the deterioration of glucose tolerance in Caucasian subjects: results of the Paris Prospective Study. Diabetologia. 1997;40(9):1101-6.

8. Laaksonen DE, Lakka TA, Lakka HM, Nyyssonen K, Rissanen T, Niskanen LK et al. Serum fatty acid composition predicts development of impaired fasting glycaemia and diabetes in middle-aged men. Diabetic Medicine. 2002;19(6):456-64.

9. Houston D, Ding J, Lee J, Garcia M, Kanaya A, Tylavsky F et al. Dietary fat and cholesterol and risk of cardiovascular disease in older adults: The Health ABC Study. Nutrition Metabolism and Cardiovascular Diseases. 2011;21(6):430-7.

10. Otto MC, Nettleton JA, Lemaitre RN, Steffen LS, Kromhout D, Rich SS et al. Biomarkers of Dairy Fatty Acids and Incidence of Cardiovascular Disease in the Multi-Ethnic Study of Atherosclerosis (MESA). Circulation. 2013;127(12).

11. Cespedes E, Baylin A, Campos H. Adipose tissue n-3 fatty acids and metabolic syndrome. European Journal of Clinical Nutrition. 2015;69(1):114-20.

12. Del Gobbo LC, Imamura F, Aslibekyan S, Marklund M, Virtanen JK, Wennberg M et al. omega-3 Polyunsaturated Fatty Acid Biomarkers and Coronary Heart Disease Pooling Project of 19 Cohort Studies. Jama Internal Medicine. 2016;176(8):1155-66.

13. Saber H, Yakoob MY, Shi P, Longstreth W, Rimm EB, Lemaitre RN et al. Circulating Phospholipid n-3 Polyunsaturated Fatty Acids and Incident Atherothrombotic and Cardioembolic Ischemic Stroke in 3 Large US Cohorts. Circulation. 2016;133.

14. Bigornia SJ, Lichtenstein AH, Harris WS, Tucker KL. Associations of erythrocyte fatty acid patterns with insulin resistance. American Journal of Clinical Nutrition. 2016;103(3):902-9.

15. Cabout M, Alssema M, Nijpels G, Stehouwer CD, Zock PL, Brouwer IA et al. Circulating linoleic acid and alpha-linolenic acid and glucose metabolism: the Hoorn Study. European Journal of Nutrition. 2017;56(6):2171-80.

16. Baghdasarian S, Lin HP, Pickering RT, Mott MM, Singer MR, Bradlee M et al. Dietary Cholesterol Intake Is Not Associated with Risk of Type 2 Diabetes in the Framingham Offspring Study. Nutrients. 2018;10(6).

17. Block RC, Liu L, Herrington DM, Huang S, Tsai MY, O'Connell TD et al. Predicting Risk for Incident Heart Failure With Omega-3 Fatty Acids From MESA. Jacc-Heart Failure. 2019;7(8):651-61.

18. Marklund M, Wu JH, Imamura F, Del Gobbo LC, Fretts A, de Goede J et al. Biomarkers of Dietary Omega-6 Fatty Acids and Incident Cardiovascular Disease and Mortality: An Individual-Level Pooled Analysis of 30 Cohort Studies. Circulation. 2019;139(21):2422-36.

19. Hu FB, Manson JE, Stampfer MJ, Colditz G, Liu S, Solomon CG et al. Diet, lifestyle, and the risk of type 2 diabetes mellitus in women. New England Journal of Medicine. 2001;345(11):790-7.

20. Jiang R, Manson JE, Stampfer MJ, Liu S, Willett WC, Hu FB. Nut and peanut butter consumption and risk of type 2 diabetes in women. JAMA. 2002;288(20):2554-60.

21. Schulze MB, Manson JE, Willett WC, Hu FB. Processed meat intake and incidence of Type 2 diabetes in younger and middle-aged women. Diabetologia. 2003;46(11):1465-73.

22. Choi HK, Willett WC, Stampfer MJ, Rimm E, Hu FB. Dairy consumption and risk of type 2 diabetes mellitus in men - A prospective study. Archives of Internal Medicine. 2005;165(9):997-1003.

23. Liu S, Choi HK, Ford E, Song Y, Klevak A, Buring JE et al. A prospective study of dairy intake and the risk of type 2 diabetes in women. Diabetes Care. 2006;29(7):1579-84.

24. Schulze MB, Schulz M, Heidemann C, Schienkiewitz A, Hoffmann K, Boeing H. Carbohydrate intake and incidence of type 2 diabetes in the European Prospective Investigation into Cancer and Nutrition (EPIC)-Potsdam Study. British Journal of Nutrition. 2008;99(5):1107-16.

25. Djousse L, Kamineni A, Nelson TL, Carnethon M, Mozaffarian D, Siscovick D et al. Egg consumption and risk of type 2 diabetes in older adults. AmJ ClinNutr. 2010;92(2):422-7.

26. Djousse L, Gaziano J, Buring JE, Lee IM. Egg Consumption and Risk of Type 2 Diabetes in Men and Women. Diabetes Care. 2009;32(2):295-300.

27. Sluijs I, Beulens JW, van der AD, Spijkerman AM, Grobbee DE, van der Schouw YT. Dietary intake of total, animal, and vegetable protein and risk of type 2 diabetes in the European Prospective Investigation into Cancer and Nutrition (EPIC)-NL study. Diabetes Care. 2010;33(1):43-8.

28. Margolis KL, Wei F, de Boer IH, Howard BV, Liu S, Manson JE et al. A Diet High in Low-Fat Dairy Products Lowers Diabetes Risk in Postmenopausal Women. Journal of Nutrition. 2011;141(11):1969-74.

29. Nanri A, Mizoue T, Noda M, Takahashi Y, Matsushita Y, Poudel-Tandukar K et al. Fish intake and type 2 diabetes in Japanese men and women: the Japan Public Health Center-based Prospective Study. American Journal of Clinical Nutrition. 2011;94(3):884-91.

30. Pan A, Sun Q, Bernstein AM, Schulze MB, Manson JE, Willett WC et al. Red meat consumption and risk of type 2 diabetes: 3 cohorts of US adults and an updated meta-analysis. American Journal of Clinical Nutrition. 2011;94(4):1088-96.

31. Lajous M, Tondeur L, Fagherazzi G, de Lauzon-Guillain B, Boutron-Ruaualt MC, Clavel-Chapelon F. Processed and Unprocessed Red Meat Consumption and Incident Type 2 Diabetes Among French Women. Diabetes Care. 2012;35(1):128-30.

32. Patel PS, Forouhi NG, Kuijsten A, Schulze MB, van Woudenbergh GJ, Ardanaz E et al. The prospective association between total and type of fish intake and type 2 diabetes in 8 European countries: EPIC-InterAct Study. American Journal of Clinical Nutrition. 2012;95(6):1445-53.

33. Abiemo EE, Alonso A, Nettleton JA, Steffen LM, Bertoni AG, Jain A et al. Relationships of the Mediterranean dietary pattern with insulin resistance and diabetes incidence in the Multi-Ethnic Study of Atherosclerosis (MESA). British Journal of Nutrition. 2013;109(8):1490-7.

34. Mozaffarian D, Otto MC, Lemaitre RN, Fretts AM, Hotamisligil G, Tsai MY et al. trans-Palmitoleic acid, other dairy fat biomarkers, and incident diabetes: the Multi-Ethnic Study of Atherosclerosis (MESA). American Journal of Clinical Nutrition. 2013;97(4):854-61.

35. Oba S, Nanri A, Kurotani K, Goto A, Kato M, Mizoue T et al. Dietary glycemic index, glycemic load and incidence of type 2 diabetes in Japanese men and women: the Japan Public Health Center-based Prospective Study. NutrJ. 2013;12(1):165.

36. Pan A, Sun Q, Manson JE, Willett WC, Hu FB. Walnut Consumption Is Associated with Lower Risk of Type 2 Diabetes in Women. Journal of Nutrition. 2013;143(4):512-8.

37. Cahill LE, Pan A, Chiuve SE, Sun Q, Willett WC, Hu FB et al. Fried-food consumption and risk of type 2 diabetes and coronary artery disease: a prospective study in 2 cohorts of US women and men. American Journal of Clinical Nutrition. 2014;100(2):667-75.

38. Koloverou E, Panagiotakos DB, Pitsavos C, Chrysohoou C, Georgousopoulou EN, Pitaraki E et al. 10-year incidence of diabetes and associated risk factors in Greece: the ATTICA study (2002-2012). RevDiabetStud. 2014;11(2):181-9.

39. Kurotani K, Nanri A, Goto A, Mizoue T, Noda M, Oba S et al. Cholesterol and egg intakes and the risk of type 2 diabetes: The Japan Public Health Center-based Prospective Study. British Journal of Nutrition. 2014;112(10):1636-43.

40. O'Connor LM, Lentjes MA, Luben RN, Khaw KT, Wareham NJ, Forouhi NG. Dietary dairy product intake and incident type 2 diabetes: a prospective study using dietary data from a 7-day food diary. Diabetologia. 2014;57(5):909-17.

41. Rylander C, Sandanger TM, Engeset D, Lund E. Consumption of Lean Fish Reduces the Risk of Type 2 Diabetes Mellitus: A Prospective Population Based Cohort Study of Norwegian Women. Plos One. 2014;9(2).

42. Virtanen JK, Mursu J, Voutilainen S, Uusitupa M, Tuomainen TP. Serum Omega-3 Polyunsaturated Fatty Acids and Risk of Incident Type 2 Diabetes in Men: The Kuopio Ischemic Heart Disease Risk Factor Study. Diabetes Care. 2014;37(1):189-96.

43. Buijsse B, Boeing H, Drogan D, Schulze M, Feskens E, Amiano P et al. Consumption of fatty foods and incident type 2 diabetes in populations from eight European countries. European Journal of Clinical Nutrition. 2015;69(4):455-61.

44. Guasch-Ferre M, Hruby A, Salas-Salvado J, Martinez-Gonzalez MA, Sun Q, Willett WC et al. Olive oil consumption and risk of type 2 diabetes in US women. American Journal of Clinical Nutrition. 2015;102(2):479-86.

45. Lajous M, Bijon A, Fagherazzi G, Balkau B, Boutron-Ruault MC, Clavel-Chapelon F. Egg and cholesterol intake and incident type 2 diabetes among French women. British Journal of Nutrition. 2015;114(10):1667-73.

46. Brouwer-Brolsma E, van Woudenbergh G, Elferink S, Singh-Povel C, Hofman A, Dehghan A et al. Intake of different types of dairy and its prospective association with risk of type 2 diabetes: The Rotterdam Study. Nutrition Metabolism and Cardiovascular Diseases. 2016;26(11):987-95.

47. Diaz-Lopez A, Bullo M, Martinez-Gonzalez MA, Corella D, Estruch R, Fito M et al. Dairy product consumption and risk of type 2 diabetes in an elderly Spanish Mediterranean population at high cardiovascular risk. European Journal of Nutrition. 2016;55(1):349-60.

48. Mari-Sanchis A, Gea A, Basterra-Gortari FJ, Martinez-Gonzalez MA, Beunza JJ, Bes-Rastrollo M. Meat Consumption and Risk of Developing Type 2 Diabetes in the SUN Project: A Highly Educated Middle-Class Population. PLoS One. 2016;11(7):e0157990.

49. Pastorino S, Richards M, Pierce M, Ambrosini GL. A high-fat, high-glycaemic index, low-fibre dietary pattern is prospectively associated with type 2 diabetes in a British birth cohort. BrJ Nutr. 2016;115(9):1632-42.

50. Satija A, Bhupathiraju SN, Rimm EB, Spiegelman D, Chiuve SE, Borgi L et al. Plant-Based Dietary Patterns and Incidence of Type 2 Diabetes in US Men and Women: Results from Three Prospective Cohort Studies. Plos Medicine. 2016;13(6).

51. Akter S, Kurotani K, Sato M, Hayashi T, Kuwahara K, Matsushita Y et al. High Serum Phospholipid Dihomo-gamma-Linoleic Acid Concentration and Low Delta 5-Desaturase Activity Are Associated with Increased Risk of Type 2 Diabetes among Japanese Adults in the Hitachi Health Study. Journal of Nutrition. 2017;147(8):1558-66.

52. Asghari G, Ghorbani Z, Mirmiran P, Azizi F. Nut consumption is associated with lower incidence of type 2 diabetes: The Tehran Lipid and Glucose Study. Diabetes & Metabolism. 2017;43(1):18-24.

53. Feldman AL, Long GH, Johansson I, Weinehall L, Fhaerm E, Wennberg P et al. Change in lifestyle behaviors and diabetes risk: evidence from a population-based cohort study with 10 year follow-up. International Journal of Behavioral Nutrition and Physical Activity. 2017;14.

54. Hruby A, Ma J, Rogers G, Meigs JB, Jacques PF. Associations of Dairy Intake with Incident Prediabetes or Diabetes in Middle-Aged Adults Vary by Both Dairy Type and Glycemic Status. Journal of Nutrition. 2017;147(9):1764-75.

55. Wallin A, Di Giuseppe D, Orsini N, Akesson A, Forouhi NG, Wolk A. Fish consumption and frying of fish in relation to type 2 diabetes incidence: a prospective cohort study of Swedish men. European Journal of Nutrition. 2017;56(2):843-52.

56. Wu JHY, Marklund M, Imamura F, Tintle N, Ardisson Korat AV, de GJ et al. Omega-6 fatty acid biomarkers and incident type 2 diabetes: pooled analysis of individual-level data for 39 740 adults from 20 prospective cohort studies. Lancet Diabetes Endocrinol. 2017;5(12):965-74.

57. Simila M, Kontto J, Valsta L, Mannisto S, Albanes D, Virtamo J. Carbohydrate substitution for fat or protein and risk of type 2 diabetes in male smokers. European Journal of Clinical Nutrition. 2012;66(6):716-21.

58. Liu S, van der Schouw YT, Soedamah-Muthu SS, Spijkerman AMW, Sluijs I. Intake of dietary saturated fatty acids and risk of type 2 diabetes in the European Prospective Investigation into Cancer and Nutrition-Netherlands cohort: associations by types, sources of fatty acids and substitution by macronutrients. Eur J Nutr. 2018. doi:10.1007/s00394-018-1630-4.

59. Andre P, Balkau B, Born C, Royer B, Wilpart E, Charles MA et al. Hepatic markers and development of type 2 diabetes in middle aged men and women: a three-year follow-up study - The DESIR study (Data from an Epidemiological Study on the Insulin Resistance syndrome). Diabetes & Metabolism. 2005;31(6):542-50.

60. Montonen J, Jarvinen R, Heliovaara M, Reunanen A, Aromaa A, Knekt P. Food consumption and the incidence of type II diabetes mellitus. European Journal of Clinical Nutrition. 2005;59(3):441-8.

61. van Dam RM, Hu FB, Rosenberg L, Krishnan S, Palmer JR. Dietary calcium and magnesium, major food sources, and risk of type 2 diabetes in US black women. Diabetes Care. 2006;29(10):2238-43.

62. Halton TL, Willett WC, Liu SM, Manson JE, Stampfer MJ, Hu FB. Potato and french fry consumption and risk of type 2 diabetes in women. American Journal of Clinical Nutrition. 2006;83(2):284-90.

63. Tso AW, Xu A, Sham PC, Wat NM, Wang Y, Fong CH et al. Serum adipocyte fatty acid-binding protein as a new biomarker, predicting the development of type 2 diabetes - A 10-year prospective study in a Chinese cohort. Diabetes Care. 2007;30(10):2667-72.

64. Dehghan A, van Hoek M, Sijbrands EJ, Stijnen T, Hofman A, Witteman JC. Risk of type 2 diabetes attributable to C-reactlve protein and other risk factors. Diabetes Care. 2007;30(10):2695-9.

65. Martinez-Gonzalez M, Fuente-Arrillaga C, Nunez-Cordoba J, Basterra-Gortari F, Beunza J, Vazquez Z et al. Adherence to Mediterranean diet and risk of developing diabetes: prospective cohort study. Bmj-British Medical Journal. 2008;336(7657):1348-51.

66. Vang A, Singh PN, Lee JW, Haddad EH, Brinegar CH. Meats, processed meats, obesity, weight gain and occurrence of diabetes among adults: Findings from Adventist Health Studies. Annals of Nutrition and Metabolism. 2008;52(2):96-104.

67. Erber E, Hopping BN, Grandinetti A, Park SY, Kolonel LN, Maskarinec G. Dietary Patterns and Risk for Diabetes The Multiethnic Cohort. Diabetes Care. 2010;33(3):532-8.

68. De Koning L, Chiuve SE, Fung TT, Willett WC, Rimm EB, Hu FB. Diet-Quality Scores and the Risk of Type 2 Diabetes in Men. Diabetes Care. 2011;34(5):1150-6.

69. De Koning L, Fung TT, Liao X, Chiuve SE, Rimm EB, Willett WC et al. Low-carbohydrate diet scores and risk of type 2 diabetes in men. American Journal of Clinical Nutrition. 2011;93(4):844-50.

70. Gast GC, Spijkerman AM, van dA, Jacobs-van der Bruggen M, Verschuren W. Five-Year Changes in Biologic Risk Factors and Risk of Type 2 Diabetes: Are Attained But Not Initial Risk Factor Levels of Importance? American Journal of Epidemiology. 2012;176(8):720-5.

71. Sluijs I, Forouhi NG, Beulens JW, van der Schouw YT, Agnoli C, Arriola L et al. The amount and type of dairy product intake and incident type 2 diabetes: results from the EPIC-InterAct Study. American Journal of Clinical Nutrition. 2012;96(2):382-90.

72. van Woudenbergh GJ, Kuijsten A, Tigcheler B, Sijbrands EJ, van Rooij FJ, Hofman A et al. Meat Consumption and Its Association With C-Reactive Protein and Incident Type 2 Diabetes The Rotterdam Study. Diabetes Care. 2012;35(7):1499-505.

73. Kurotani K, Nanri A, Goto A, Mizoue T, Noda M, Oba S et al. Red meat consumption is associated with the risk of type 2 diabetes in men but not in women: a Japan Public Health Center-based Prospective Study. British Journal of Nutrition. 2013;110(10):1910-8.

74. Struijk E, Heraclides A, Witte D, Soedamah-Muthu S, Geleijnse J, Toft U et al. Dairy product intake in relation to glucose regulation indices and risk of type 2 diabetes. Nutrition Metabolism and Cardiovascular Diseases. 2013;23(9):822-8.

75. Sakurai M, Nakamura K, Miura K, Takamura T, Yoshita K, Sasaki S et al. Family history of diabetes, lifestyle factors, and the 7-year incident risk of type 2 diabetes mellitus in middle-aged Japanese men and women. Journal of Diabetes Investigation. 2013;4(3):261-8.

76. Soedamah-Muthu SS, Masset G, Verberne L, Geleijnse JM, Brunner EJ. Consumption of dairy products and associations with incident diabetes, CHD and mortality in the Whitehall II study. British Journal of Nutrition. 2013;109(4):718-26.

77. Palli D, InterAct C. Association between dietary meat consumption and incident type 2 diabetes: the EPIC-InterAct study. Diabetologia. 2013;56(1):47-59. doi:10.1007/s00125-012-2718-7.

78. Chen M, Sun Q, Giovannucci E, Mozaffarian D, Manson JE, Willett WC et al. Dairy consumption and risk of type 2 diabetes: 3 cohorts of US adults and an updated meta-analysis. Bmc Medicine. 2014;12.

79. Dominguez LJ, Bes-Rastrollo M, Javier Basterra-Gortari F, Gea A, Barbagallo M, Martinez-Gonzalez MA. Association of a Dietary Score with Incident Type 2 Diabetes: The Dietary-Based Diabetes-Risk Score (DDS). Plos One. 2015;10(11).

80. Virtanen JK, Mursu J, Tuomainen TP, Virtanen HE, Voutilainen S. Egg consumption and risk of incident type 2 diabetes in men: the Kuopio lschaemic Heart Disease Risk Factor Study. American Journal of Clinical Nutrition. 2015;101(5):1088-96.

81. Bao W, Li S, Chavarro JE, Tobias DK, Zhu Y, Hu FB et al. Low Carbohydrate-Diet Scores and Long-term Risk of Type 2 Diabetes Among Women With a History of Gestational Diabetes Mellitus: A Prospective Cohort Study. Diabetes Care. 2016;39(1):43-9.

82. Imamura F, Sharp SJ, Koulman A, Schulze MB, Kroeger J, Griffin JL et al. A combination of plasma phospholipid fatty acids and its association with incidence of type 2 diabetes: The EPIC-InterAct case-cohort study. Plos Medicine. 2017;14(10).

83. Jacobs S, Kroeger J, Schulze MB, Frank LK, Franke AA, Cheng I et al. Dietary Patterns Derived by Reduced Rank Regression Are Inversely Associated with Type 2 Diabetes Risk across 5 Ethnic Groups in the Multiethnic Cohort. Current Developments in Nutrition. 2017;1(5).

84. Drehmer M, Odegaard AO, Schmidt MI, Duncan BB, Cardoso LdO, Alvim Matos SM et al. Brazilian dietary patterns and the dietary approaches to stop hypertension (DASH) diet-relationship with metabolic syndrome and newly diagnosed diabetes in the ELSA-Brasil study. Diabetology & Metabolic Syndrome. 2017;9.

85. Farhadnejad H, Teymoori F, Asghari C, Mirmiran P, Azizi F. The Association of Potato IntakeWith Risk for Incident Type 2 Diabetes in Adults. Canadian Journal of Diabetes. 2018;42(6):613-8.

86. Arab L, Dhaliwal SK, Martin CJ, Larios AD, Jackson NJ, Elashoff D. Association between walnut consumption and diabetes risk in NHANES. Diabetes-Metabolism Research and Reviews. 2018;34(7).

87. Lee J, Kim J. Egg consumption is associated with a lower risk of type 2 diabetes in middle-aged and older men. Nutrition Research and Practice. 2018;12(5):396-405.

88. Shan R, Duan W, Liu L, Qi J, Gao J, Zhang Y et al. Low-Carbohydrate, High-Protein, High-Fat Diets Rich in Livestock, Poultry and Their Products Predict Impending Risk of Type 2 Diabetes in Chinese Individuals that Exceed Their Calculated Caloric Requirement. Nutrients. 2018;10(1).

89. Brahimaj A, Rivadeneira F, Muka T, Sijbrands EJ, Franco OH, Dehghan A et al. Novel metabolic indices and incident type 2 diabetes among women and men: the Rotterdam Study. Diabetologia. 2019;62(9):1581-90.

90. Ericson U, Brunkwall L, Dias JA, Drake I, Hellstrand S, Gullberg B et al. Food patterns in relation to weight change and incidence of type 2 diabetes, coronary events and stroke in the Malmo Diet and Cancer cohort. European Journal of Nutrition. 2019;58(5):1801-14.

91. Kummer K, Jensen PN, Kratz M, Lemaitre RN, Howard BV, Cole SA et al. Full-Fat Dairy Food Intake is Associated with a Lower Risk of Incident Diabetes Among American Indians with Low Total Dairy Food Intake. Journal of Nutrition. 2019;149(7):1238-44.

92. Khalili-Moghadam S, Mirmiran P, Bahadoran Z, Azizi F. The Mediterranean diet and risk of type 2 diabetes in Iranian population. European Journal of Clinical Nutrition. 2019;73(1):72-8.

93. Djousse L, Khawaja O, Bartz TM, Biggs ML, Ix JH, Zieman SJ et al. Plasma Fatty Add-Binding Protein 4, Nonesterified Fatty Acids, and Incident Diabetes in Older Adults. Diabetes Care. 2012;35(8):1701-7.

94. Krachler B, Norberg M, Eriksson JW, Hallmans G, Johansson I, Vessby B et al. Fatty acid profile of the erythrocyte membrane preceding development of Type 2 diabetes mellitus. Nutrition Metabolism and Cardiovascular Diseases. 2008;18(7):503-10.

95. Mozaffarian D, Cao H, King IB, Lemaitre RN, Song X, Siscovick DS et al. Trans-palmitoleic acid, metabolic risk factors, and new-onset diabetes in U.S. adults: a cohort study. Ann Intern Med. 2010;153(12):790-9. doi:10.7326/0003-4819-153-12-201012210-00005.

96. Mozaffarian D, Cao H, King IB, Lemaitre RN, Song X, Siscovick DS et al. Circulating palmitoleic acid and risk of metabolic abnormalities and new-onset diabetes. American Journal of Clinical Nutrition. 2010;92(6):1350-8.

97. Mahendran Y, Cederberg H, Vangipurapu J, Kangas AJ, Soininen P, Kuusisto J et al. Glycerol and Fatty Acids in Serum Predict the Development of Hyperglycemia and Type 2 Diabetes in Finnish Men. Diabetes Care. 2013;36(11):3732-8.

98. Santaren ID, Watkins SM, Liese AD, Wagenknecht LE, Rewers MJ, Haffner SM et al. Serum pentadecanoic acid (15:0), a short-term marker of dairy food intake, is inversely associated with incident type 2 diabetes and its underlying disorders. American Journal of Clinical Nutrition. 2014;100(6):1532-40.

99. Forouhi NG, Koulman A, Sharp SJ, Imamura F, Kroger J, Schulze MB et al. Differences in the prospective association between individual plasma phospholipid saturated fatty acids and incident type 2 diabetes: the EPIC-InterAct case-cohort study. Lancet Diabetes & Endocrinology. 2014;2(10):810-8.

100. Alhazmi A, Stojanovski E, Garg ML, McEvoy M. Fasting Whole Blood Fatty Acid Profile and Risk of Type 2 Diabetes in Adults: A Nested Case Control Study. Plos One. 2014;9(5).

101. Ahmadi-Abhari S, Luben RN, Powell N, Bhaniani A, Chowdhury R, Wareham NJ et al. Dietary intake of carbohydrates and risk of type 2 diabetes: the European Prospective Investigation into Cancer-Norfolk study. British Journal of Nutrition. 2014;111(2):342-52.

102. Mahendran Y, Agren J, Uusitupa M, Cederberg H, Vangipurapu J, Stancakova A et al. Association of erythrocyte membrane fatty acids with changes in glycemia and risk of type 2 diabetes. American Journal of Clinical Nutrition. 2014;99(1):79-85.

103. Zong G, Sun Q, Yu D, Zhu J, Sun L, Ye X et al. Dairy Consumption, Type 2 Diabetes, and Changes in Cardiometabolic Traits: A Prospective Cohort Study of Middle-Aged and Older Chinese in Beijing and Shanghai. Diabetes Care. 2014;37(1):56-63.

104. Lankinen MA, Stancakova A, Uusitupa M, Agren J, Pihlajamaki J, Kuusisto J et al. Plasma fatty acids as predictors of glycaemia and type 2 diabetes. Diabetologia. 2015;58(11):2533-44.

105. Lemaitre RN, Fretts AM, Sitlani CM, Biggs ML, Mukamal K, King IB et al. Plasma phospholipid very-long-chain saturated fatty acids and incident diabetes in older adults: the Cardiovascular Health Study. American Journal of Clinical Nutrition. 2015;101(5):1047-54.

106. Lu Y, Wang Y, Ong CN, Subramaniam T, Choi HW, Yuan JM et al. Metabolic signatures and risk of type 2 diabetes in a Chinese population: an untargeted metabolomics study using both LC-MS and GC-MS. Diabetologia. 2016;59(11):2349-59.

107. Forouhi NG, Imamura F, Sharp SJ, Koulman A, Schulze MB, Zheng J et al. Association of Plasma Phospholipid n-3 and n-6 Polyunsaturated Fatty Acids with Type 2 Diabetes: The EPIC-InterAct Case-Cohort Study. Plos Medicine. 2016;13(7).

108. Santaren ID, Bazinet RP, Liu Z, Johnston LW, Retnakaran R, Harris SB et al. Serum Fatty Acids Derived from Dairy Consumption Are Associated with Insulin Sensitivity and Beta-Cell Function: The Prospective Metabolism and Islet Cell Evaluation (PROMISE) Cohort. Diabetes. 2016;65:A95-A.

109. Yary T, Voutilainen S, Tuomainen TP, Ruusunen A, Nurmi T, Virtanen JK. Serum n-6 polyunsaturated fatty acids, Delta 5-and Delta 6-desaturase activities, and risk of incident type 2 diabetes in men: the Kuopio Ischaemic Heart Disease Risk Factor Study. American Journal of Clinical Nutrition. 2016;103(5):1337-43.

110. Yakoob MY, Shi P, Willett WC, Rexrode KM, Campos H, Orav EJ et al. Circulating Biomarkers of Dairy Fat and Risk of Incident Diabetes Mellitus Among Men and Women in the United States in Two Large Prospective Cohorts. Circulation. 2016;133(17):1645-54.

111. Takkunen MJ, Schwab US, de Mello VD, Eriksson JG, Lindstrom J, Tuomilehto J et al. Longitudinal associations of serum fatty acid composition with type 2 diabetes risk and markers of insulin secretion and sensitivity in the Finnish Diabetes Prevention Study. European Journal of Nutrition. 2016;55(3):967-79.

112. Harris WS, Luo J, Pottala JV, Margolis KL, Espeland MA, Robinson JG. Red Blood Cell Fatty Acids and Incident Diabetes Mellitus in the Women's Health Initiative Memory Study. Plos One. 2016;11(2).

113. Savolainen O, Lind MV, Bergstrom G, Fagerberg B, Sandberg AS, Ross A. Biomarkers of food intake and nutrient status are associated with glucose tolerance status and development of type 2 diabetes in older Swedish women. American Journal of Clinical Nutrition. 2017;106(5):1302-10.

114. Imamura F, Fretts A, Marklund M, Korat AV, Yang WS, Lankinen M et al. Fatty acid biomarkers of dairy fat consumption and incidence of type 2 diabetes: A pooled analysis of prospective cohort studies. Plos Medicine. 2018;15(10).

115. Lin Js, Dong Hl, Chen Gd, Chen Zy, Dong Xw, Zheng JS et al. Erythrocyte Saturated Fatty Acids and Incident Type 2 Diabetes in Chinese Men and Women: A Prospective Cohort Study. Nutrients. 2018;10(10).

116. Wang Y, Meng X, Deng X, Okekunle AP, Wang P, Zhang Q et al. Postprandial Saturated Fatty Acids Increase the Risk of Type 2 Diabetes: A Cohort Study in a Chinese Population. Journal of Clinical Endocrinology & Metabolism. 2018;103(4):1438-46.

117. Lu Y, Wang Y, Zou L, Liang X, Ong CN, Tavintharan S et al. Serum Lipids in Association With Type 2 Diabetes Risk and Prevalence in a Chinese Population. Journal of Clinical Endocrinology & Metabolism. 2018;103(2):671-80.

118. Fretts AM, Imamura F, Marklund M, Micha R, Wu JHY, Murphy RA et al. Associations of circulating very-long-chain saturated fatty acids and incident type 2 diabetes: a pooled analysis of prospective cohort studies. Am J Clin Nutr. 2019;109(4):1216-23. doi:10.1093/ajcn/nqz005.

119. Pankow JS, Duncan BB, Schmidt MI, Ballantyne CM, Couper D, Hoogeveen RC et al. Fasting plasma free fatty acids and risk of type 2 diabetes - The atherosclerosis risk in communities study. Diabetes Care. 2004;27(1):77-82.

120. Steffen BT, Steffen LM, Zhou X, Ouyang P, Weir NL, Tsai MY. n-3 Fatty Acids Attenuate the Risk of Diabetes Associated With Elevated Serum Nonesterified Fatty Acids: The Multi-Ethnic Study of Atherosclerosis. Diabetes Care. 2015;38(4):575-80.

121. Almoosawi S, Prynne C, Hardy R, Stephen A. Diurnal eating rhythms: Association with long-term development of diabetes in the 1946 British birth cohort. Nutrition Metabolism and Cardiovascular Diseases. 2013;23(10):1025-30.

122. Mandalazi E, Drake I, Wirfalt E, Orho-Melander M, Sonestedt E. A High Diet Quality Based on Dietary Recommendations Is Not Associated with Lower Incidence of Type 2 Diabetes in the Malmo Diet and Cancer Cohort. International Journal of Molecular Sciences. 2016;17(6).

123. Lindstrom J, Ilanne-Parikka P, Peltonen M, Aunola S, Eriksson JG, Hemio K et al. Sustained reduction in the incidence of type 2 diabetes by lifestyle intervention: follow-up of the Finnish Diabetes Prevention Study. Lancet. 2006;368(9548):1673-9.

124. Forouhi NG. Association between consumption of dairy products and incident type 2 diabetes-insights from the European Prospective Investigation into Cancer study. Nutrition Reviews. 2015;73:15-22.

125. Spiller S, Blueher M, Hoffmann R. Plasma levels of free fatty acids correlate with type 2 diabetes mellitus. Diabetes Obesity & Metabolism. 2018;20(11):2661-9.

126. Howard BV, Aragaki AK, Tinker LF, Allison M, Hingle MD, Johnson KC et al. A Low-Fat Dietary Pattern and Diabetes: A Secondary Analysis From the Women's Health Initiative Dietary Modification Trial. Diabetes Care. 2018;41(4):680-7.

127. Kamleh MA, McLeod O, Checa A, Baldassarre D, Veglia F, Gertow K et al. Increased Levels of Circulating Fatty Acids Are Associated with Protective Effects against Future Cardiovascular Events in Nondiabetics. Journal of Proteome Research. 2018;17(2):870-8.

128. Wang L, Folsom AR, Zheng ZJ, Pankow JS, Eckfeldt JH. Plasma fatty acid composition and incidence of diabetes in middle-aged adults: the Atherosclerosis Risk in Communities (ARIC) Study. American Journal of Clinical Nutrition. 2003;78(1):91-8.

129. Harding AH, Day NE, Khaw KT, Bingham S, Luben R, Welsh A et al. Dietary fat and the risk of clinical type 2 diabetes - The European Prospective Investigation of Cancer-Norfolk study. American Journal of Epidemiology. 2004;159(1):73-82.

130. Thanopoulou AC, Karamanos BG, Angelico FV, Assaad-Khalil SH, Barbato AF, Del Ben MP et al. Dietary fat intake as risk factor for the development of diabetes - Multinational, multicenter study of the Mediterranean Group for the Study of Diabetes (MGSD). Diabetes Care. 2003;26(2):302-7.

131. Lofvenborg JE, Andersson T, Carlsson PO, Dorkhan M, Groop L, Martinell M et al. Fatty fish consumption and risk of latent autoimmune diabetes in adults. NutrDiabetes. 2014;4:e139.

132. Jo S, An WS, Park Y. Erythrocyte n-3 Polyunsaturated Fatty Acids and the Risk of Type 2 Diabetes in Koreans: A Case-Control Study. Annals of Nutrition and Metabolism. 2013;63(4):283-90.

133. Abbott KA, Veysey M, Lucock M, Niblett S, King K, Burrows T et al. Sex-dependent association between erythrocyte n-3 PUFA and type 2 diabetes in older overweight people. British Journal of Nutrition. 2016;115(8):1379-86.

134. Howard BV. Dietary fatty acids, insulin resistance, and diabetes. Lipids and Syndromes of Insulin Resistance: from Molecular Biology to Clinical Medicine. 1997;827:215-20.

135. Stoeckli R, Keller U. Nutritional fats and the risk of type 2 diabetes and cancer. Physiology & Behavior. 2004;83(4):611-5.

136. Fretts AM, Howard BV, McKnight B, Duncan GE, Beresford SA, Mete M et al. Very Long Chain Saturated Fatty Acids and Diabetes Risk: Meta-Analysis of Cohort Studies in the FORCE Consortium. American Journal of Clinical Nutrition. 2012;95(3):752-8.

137. Huang L, Lin Js, Aris IM, Yang G, Chen WQ, Li LJ. Circulating Saturated Fatty Acids and Incident Type 2 Diabetes: A Systematic Review and Meta-Analysis. Nutrients. 2019;11(5).

138. Kromhout D, Feskens EJ. Nutrition and diabetes: the role of fat. Acta Cardiol. 1993;48(5):444-5.

139. Asao K, Guallar E, Guallar-Castillon P, Bang H, Steffen LM, Folsom AR et al. A prospective study of dietary fatty acids and diabetes: The Atherosclerosis Risk in Communities (ARIC) Study. Diabetes. 2005;54:A251-A.

140. Imamura F, Lemaitre RN, King IB, Song X, Siscovick DS, Mozaffarian D. Circulating Fatty Acid Patterns and Incidence of Type 2 Diabetes Mellitus: The Cardiovascular Health Study. Circulation. 2013;127(12).

141. Marklund M, Laguzzi F, Vikstrom M, Alsharari Z, Sjogren P, Gigante B et al. Polyunsaturated Fat Intake Estimated by Circulating Biomarkers is Inversely Associated with Cardiovascular Disease and All-Cause Mortality in a Large Population-Based Cohort of Swedish Women and Men. Circulation. 2014;130.

142. Ericson U, Hellstrand S, Brunkwall L, Sonestedt E, Wallstrom P, Gullberg B et al. Food sources of fat may clarify the earlier inconsistent role of dietary fat intake for incidence of type 2 diabetes. Diabetologia. 2014;57:S32-S.

143. Dow C, Mangin M, Balkau B, Affret A, Boutron-Ruault M, Clavel-Chapelon F et al. Fatty acid consumption and incident type 2 diabetes: evidence from the E3N cohort study. Diabetologia. 2016;59:S145-S.

144. Fretts A, Imamura F, Yu C, Frazier-Wood AC, Lankinen M, Rajaobelina K et al. Very Long Chain Saturated Fatty Acids and Diabetes Risk: Meta-Analysis of Cohort Studies in the FORCE Consortium. Circulation. 2017;135.

145. Wu JH, Marklund M, Imamura F, Tintle N, Korat AV, de Goede J et al. Omega-6 Fatty Acid Biomarkers and Incident Type 2 Diabetes: A Pooled Analysis of 20 Cohort Studies. Circulation. 2017;135.

146. Merino J, Guasch-Ferre M, Ellervik C, Dashti H, Smith C, Kilpelainen T et al. Dietary fat quality and genetic risk of type 2 diabetes. Diabetologia. 2018;61:S118-S.

147. Ahola-Olli AV, Mustelin L, Kalimeri M, Kettunen J, Jokelainen JJ, Auvinen J et al. Circulating Metabolites and the Risk of Type 2 Diabetes-A Prospective Study of 10,938 Young Adults from Four Finnish Cohorts. Diabetes. 2018;67.

148. Weir NL, Johnson L, Guan W, Steffen B, Djousse L, Mukamal KJ et al. Cis-Vaccenic Acid Is Associated with Lower HOMA-IR and Incident T2D in Participants from the MESA Cohort. Diabetes. 2018;67.

149. Weir NL, Guan W, Steffen B, Steffen LM, Karger AB, Tsai MY. Omega-6 Eicosadienoic Acid Is Associated with Lower HOMA-IR and Incident T2D in Participants from the MESA Cohort. Diabetes. 2018;67.

150. Korat AA, Hu FB, Sun Q. Dairy Fat Intake and Risk of Type 2 Diabetes in 3 Cohorts of US Adults. Circulation. 2018;137.

151. Zong G, Liu G, Wanders AJ, Alssema M, Zock PL, Willett W et al. Dietary Linoleic Acid Intake Is Inversely Associated With Type 2 Diabetes Risk In Three Large Prospective Cohort Studies Of U.s. Men And Women. Circulation. 2018;137.

152. Qian F, Korat AV, Imamura F, Marklund M, Tintle N, Virtanen JK et al. Omega-3 Fatty Acid Biomarkers and Incident Type 2 Diabetes: An Individual Participant-level Pooling Project of 20 Prospective Cohort Studies. Circulation. 2019;139.

153. Qian F, Zong G, Li Y, Sun Q. Monounsaturated Fatty Acids From Plant or Animal Sources and Risk of Type 2 Diabetes in Three Large Prospective Cohorts of Men and Women. Circulation. 2019;139.

154. Bradley CA. Omega-6 Pufas and T2Dm. Nature Reviews Endocrinology. 2017;13(12).

155. Colditz GA, Manson JE, Stampfer MJ, Rosner B, Willett WC, Speizer FE. Diet and risk of clinical diabetes in women. Am J Clin Nutr. 1992;55(5):1018-23. doi:10.1093/ajcn/55.5.1018.

156. Ericson U, Sonestedt E, Gullberg B, Hellstrand S, Hindy G, Wirfalt E et al. High intakes of protein and processed meat associate with increased incidence of type 2 diabetes. BrJ Nutr. 2013;109(6):1143-53.

157. Abhari SA, Luben R, Powell N, Bhaniani A, Wareham N, Forouhi N et al. Dietary intake of carbohydrates and risk of type 2 diabetes: European Prospective Investigation into Cancer in Norfolk study. Diabetologia. 2013;56:S159-S60.

158. Patel P, Sharp S, Jansen E, Luben R, Khaw KT, Wareham N et al. Fatty acids measured in plasma and erythrocyte-membrane phospholipids and derived by food-frequency questionnaire and the risk of new-onset type 2 diabetes: a pilot study in the European Prospective Investigation into Cancer and Nutrition (EPIC)-Norfolk cohort (vol 92, pg 1214, 2010). American Journal of Clinical Nutrition. 2013;98(1):255-8.

159. Harris WS, Luo J, Pottala JV, Margolis KL, Espeland MA, Robinson JG. Red Blood Cell Fatly Acids and Incident Diabetes Mellitus in the Women's Health Initiative Memory Study. Faseb Journal. 2016;30.

160. Salmeron J, Manson JE, Stampfer MJ, Colditz GA, Wing AL, Willett WC. Dietary fiber, glycemic load, and risk of non-insulin-dependent diabetes mellitus in women. Jama-Journal of the American Medical Association. 1997;277(6):472-7.

161. Gaeini Z, Bahadoran Z, Mirmiran P, Djazayery A. The Association between Dietary Fat Pattern and the Risk of Type 2 Diabetes. Prev Nutr Food Sci. 2019;24(1):1-7. doi:10.3746/pnf.2019.24.1.1.

162. Halton TL, Liu S, Manson JE, Hu FB. Low-carbohydrate-diet score and risk of type 2 diabetes in women. Am J Clin Nutr. 2008;87(2):339-46. doi:10.1093/ajcn/87.2.339.

163. Hodge AM, English DR, O'Dea K, Sinclair AJ, Makrides M, Gibson RA et al. Plasma phospholipid and dietary fatty acids as predictors of type 2 diabetes: interpreting the role of linoleic acid. Am J Clin Nutr. 2007;86(1):189-97. doi:10.1093/ajcn/86.1.189.

164. Long G, Johansson I, Rolandsson O, Wennberg P, Fharm E, Weinehall L et al. Healthy behaviours and 10-year incidence of diabetes: A population cohort study. Preventive Medicine. 2015;71:121-7.

165. Mirmiran P, Esfandyari S, Moghadam SK, Bahadoran Z, Azizi F. Fatty acid quality and quantity of diet and risk of type 2 diabetes in adults: Tehran Lipid and Glucose Study. J Diabetes Complications. 2018;32(7):655-9. doi:10.1016/j.jdiacomp.2018.05.003.

166. Patel PS, Sharp SJ, Jansen E, Luben RN, Khaw KT, Wareham NJ et al. Fatty acids measured in plasma and erythrocyte-membrane phospholipids and derived by food-frequency questionnaire and the risk of new-onset type 2 diabetes a pilot study in the European Prospective Investigation into Cancer and Nutrition (EPIC)-Norfolk cohort. American Journal of Clinical Nutrition. 2010;92(5):1214-22.
