# Supplementary material for: Intake of dietary fats and fatty acids and the incidence of type 2 diabetes: A systematic review and dose-response meta-analysis of prospective observational studies
Source: PLoS Med. 2020 Dec 2;17(12):e1003347. doi: 10.1371/journal.pmed.1003347 (PMC7710077; doi:10.1371/journal.pmed.1003347)
Supplement: S3 Table — (DOCX) [file pmed.1003347.s011.docx]

**S3 Table**: Study characteristics of the included studies

| **Study (author, year, location, cohort)** | **Follow-up** | **Exposure assessment** | **Number of participants, sex, age** | **Total cases** | **T2D assessment** | **Exposure** | **Categories** | **RR (95% CI), HR [95% CI]** | **Adjustment factors** |
| --- | --- | --- | --- | --- | --- | --- | --- | --- | --- |
| Alhazmi, 2013,  Australia,  Australian Longitudinal Study on Women’s Health (ALSWH)  SRef (1) | 6 years | Validated dietary Questionnaire for  Epidemiological Studies (DQES) version 2 | 8370, w, 45-50 years | 311 | Self-report, validated by linking to Medicare (MBS) and  Pharmaceutical Benefits Scheme (PBS) databases for the  years 2002–2005. | Total fat | 33.87 g/d (REF)  47.47 g/d  58.99 g/d  71.93 g/d  95.51 g/d | 1.00  1.21 (0.81, 1.82)  1.12 (0.74, 1.69)  1.33 (0.88, 2.02)  1.27 (0.84, 1.91) | Area of residence, education, current smoking status, physical activity, self-rated health as good, menopausal status, BMI, alcohol consumption, total energy intake (kJ/d), SFA and MUFA intakes for total carbohydrate, SFA, MUFA and fibre intakes for total  protein, and fibre intake for total fat. |
|  |  |  |  |  |  | SFA | 12.37 g/d (REF)  17.95 g/d  22.81 g/d  29.01 g/d  40.01 g/d | 1.00  1.15 (0.79, 1.68)  1.22 (0.84, 1.76)  0.98 (0.67, 1.43)  0.71 (0.47, 1.07) |  |
|  |  |  |  |  |  | MUFA | 11.69 g/d (REF)  16.43 g/d  20.58 g/d  25.32 g/d  34.32 g/d | 1.00  1.29 (0.85, 1.94)  1.18 (0.76, 1.84)  1.30 (0.83, 2.02)  1.64 (1.06, 2.54) |  |
|  |  |  |  |  |  | PUFA | 4.31 g/d (REF)  6.77 g/d  9.15 g/d  11.95 g/d  16.52 g/d | 1.00  0.81 (0.53, 1.25)  1.10 (0.73, 1.67)  1.07 (0.71, 1.61)  1.27 (0.84, 1.90) |  |
|  |  |  |  |  |  | Total omega-3 | 0.61 g/d (REF)  0.86 g/d  1.08 g/d  1.37 g/d  1.97 g/d | 1.00  0.98 (0.63, 1.52)  1.27 (0.84, 1.92)  1.44 (0.97, 2.16)  1.55 (1.03, 2.32) |  |
|  |  |  |  |  |  | EPA+DHA | 0.09 g/d (REF)  0.17 g/d  0.25 g/d  0.38 g/d  0.73 g/d | 1.00  1.07 (0.71. 1.60)  1.16 (0.77, 1.75)  1.12 (0.75, 1.68)  1.23 (0.84, 1.80) |  |
|  |  |  |  |  |  | EPA | 0.02 g/d (REF)  0.04 g/d  0.07 g/d  0.12 g/d  0.24 g/d | 1.00  1.06 (0.71. 1.59)  1.19 (0.79, 1.79)  1.07 (0.71, 1.62)  1.24 (0.85, 1.82) |  |
|  |  |  |  |  |  | DHA | 0.06 g/d (REF)  0.11 g/d  0.17 g/d  0.26 g/d  0.49 g/d | 1.00  1.04 (0.69, 1.55)  1.07 (0.71, 1.61)  1.10 (0.73, 1.64)  1.19 (0.81, 1.74) |  |
|  |  |  |  |  |  | ALA | 0.42 g/d (REF)  0.61 g/d  0.78 g/d  0.98 g/d  1.40 g/d | 1.00  1.20 (0.78, 1.82)  1.17 (0.76, 1.80)  1.32 (0.86, 2.01)  1.84 (1.25, 2.71) |  |
|  |  |  |  |  |  | Total omega-6 | 3.54 g/d (REF)  5.47 g/d  7.43 g/d  9.86 g/d  13.87 g/d | 1.00  1.25 (0.83, 1.90)  1.18 (0.76, 1.83)  1.28 (0.82, 1.99)  1.60 (1.03, 2.48) |  |
|  |  |  |  |  |  | Omega-6:omega-3 - ratio | 0.09 (REF)  0.12  0.15  0.19  0.25 | 1.00  1.15 (0.79, 1.66)  1.07 (0.73, 1.57)  0.71 (0.47, 1.10)  1.14 (0.80, 1.64) |  |
| Brostow, 2011, China, Singapore Chinese Health Study (SCHS) SRef (2) | 5.7 years | Validated, semi-quantitative FFQ including 165 commonly consumed items | 43,176, m/w, 45-74 years | 2252 | Self-reported, validation study of incident diabetes mellitus cases | Total omega-3 | 0.45 g/d (REF) | 1.00 | Age, sex, dialect, year of interview, educational level, BMI, physical activity, smoking status, alcohol use, hypertension, intakes of omega-6 or omega-3, MUFA, SFA, dietary fiber, protein, and total energy |
|  |  |  |  |  |  |  | 0.66 g/d | 0.87 [0.75, 1.00] |  |
|  |  |  |  |  |  |  | 0.82 g/d | 0.88 [0.76, 1.02] |  |
|  |  |  |  |  |  |  | 1.02 g/d | 0.80 [0.68, 0.94] |  |
|  |  |  |  |  |  |  | 1.54 g/d | 0.78 [0.65, 0.94] |  |
|  |  |  |  |  |  | Total omega-6 | 3.50 g/d (REF) | 1.00 |  |
|  |  |  |  |  |  |  | 5.40 g/d | 0.94 [0.81, 1.08] |  |
|  |  |  |  |  |  |  | 7.10 g/d | 1.00 [0.87, 1.17] |  |
|  |  |  |  |  |  |  | 9.30 g/d | 0.91 [0.78, 1.07] |  |
|  |  |  |  |  |  |  | 14.60 g/d | 0.93 [0.87, 1.12] |  |
|  |  |  |  |  |  | Omega-6:omega-3 - ratio | 5.90 (REF) | 1.00 |  |
|  |  |  |  |  |  |  | 7.20 | 0.93 [0.81, 1.06] |  |
|  |  |  |  |  |  |  | 8.30 | 1.08 [0.94, 1.24] |  |
|  |  |  |  |  |  |  | 10.00 | 1.03 [0.89, 1.20] |  |
|  |  |  |  |  |  |  | 14.10 | 0.98 [0.85, 1.14] |  |
|  |  |  |  |  |  | EPA&DHA | 0.11 g/d (REF) | 1.00 |  |
|  |  |  |  |  |  |  | 0.22 g/d | 1.01 [0.88, 1.17] |  |
|  |  |  |  |  |  |  | 0.30 g/d | 0.99 [0.85, 1.14] |  |
|  |  |  |  |  |  |  | 0.38 g/d | 0.94 [0.80, 1.10] |  |
|  |  |  |  |  |  |  | 0.60 g/d | 0.93 [0.77, 1.11] |  |
|  |  |  |  |  |  | ALA | 0.27 g/d (REF) | 1.00 |  |
|  |  |  |  |  |  |  | 0.40 g/d | 0.91 [0.80, 1.04] |  |
|  |  |  |  |  |  |  | 0.51 g/d | 0.81 [0.70, 0.93] |  |
|  |  |  |  |  |  |  | 0.65 g/d | 0.78 [0.67, 0.90] |  |
|  |  |  |  |  |  |  | 1.06 g/d | 0.79 [0.67, 0.93] |  |
| Djoussé, 2011, USA, Womens’ Health Study (WHS)  SRef (3) | 12.4 years | Validated baseline 128-FFQ | 36,328, w, 54.6 years | 2370 | Self-report, validated using the ADA criteria (additional information via telephone interview and supplemental questionnaire) | ALA | 0.79 g/d (REF)  0.96 g/d  1.11 g/d  1.29 g/d  1.59 g/d | 1.00  0.94 [0.82, 1.09]  0.98 [0.85, 1.14]  1.00 [0.86, 1.17]  1.01 [0.85, 1.21] | Age, BMI, parental history of diabetes, smoking, exercise, alcohol intake, menopausal state, red-meat intake, quintiles of  energy intake, linoleic acid, a-linolenic acid, dietary magnesium, trans fat, saturated fat, cereal fiber, and glycemic index |
|  |  |  |  |  |  | EPA | 0.01 g/d (REF)  0.02 g/d  0.03 g/d  0.08 g/d  0.12 g/d | 1.00  1.08 [0.94, 1.24]  1.25 [1.11, 1.42]  1.30 [1.13, 1.49]  1.38 [1.21, 1.59] |  |
|  |  |  |  |  |  | DHA | 0.04 g/d (REF)  0.09 g/d  0.12 g/d  0.17 g/d  0.17 g/d | 1.00  1.21 [1.06, 1.38]  1.21 [1.06, 1.39]  1.46 [1.28, 1.68]  1.52 [1.33, 1.75] |  |
|  |  |  |  |  |  | Marine-n3 | 0.07 g/d (REF)  0.13 g/d  0.18 g/d  0.28 g/d  0.43 g/d | 1.00  1.17 [1.03, 1.33]  1.20 [1.05, 1.38]  1.46 [1.28, 1.66]  1.44 [1.25, 1.65] |  |
| Djoussé, 2011, USA, Cardiovascular Health Study (CHS)  SRef (4) | 9.6 years | Validated picture-sort FFQ in 1989–1990 and a FFQ (1995–1996 examination) | 3088, m/w, 75.6 years for men, 74.7 years for woemen | 204 | 1) the new use of insulin or oral hypoglycemic agents, 2) a  fasting glucose concentration ≥7 mmol/L (126 mg/dL), or 3) a  nonfasting glucose concentration ≥11.1 mmol/L (200 mg/dL). | EPA+DHA | 0.105 g/d (REF)  0.235 g/d  0.430 g/d  0.690 g/d | 1.00  1.11 (0.74, 1.66)  0.78 (0.50, 1.22)  1.04 (0.67, 1.60) | Age, race (black or nonblack), sex, clinic site, BMI, alcohol consumption, physical  activity, current smoking, LDL cholesterol, and linoleic acid |
|  |  |  |  |  |  | ALA | 0.095 g/d (REF)  0.125 g/d  0.160 g/d  0.200 g/d | 1.00  0.82 (0.51, 1.33)  0.99 (0.56, 1.77)  0.50 (0.24, 1.05) |  |
| Dow, 2016, France, E3N (Etude Epidémiologique auprès des femmes de la Mutuelle  Générale de l’Education Nationale) (EPIC-France)  SRef (5) | 18 years | Validated 208-item FFQ | 71,334, w, 52.9 years | 2610 | self-report or  reimbursements  from health insurance records at least once between January  2004 and March 2012  Additional questionnaire -> cases validated, if one of the following criteria was met: 1) fasting plasma glucose ≥7·0 mmol/l, 2 ) random glucose  ≥11·1 mmol/l at diagnosis, 3) report of diabetic medication use. 4)  or last values of fasting glucose or HbA1c concentrations  ≥7·0 mmol/l or ≥7%, respectively | SFA | <33.3 g/d (REF)  33.3-38.9 g/d  ≥38.9 g/d | 1.00  1.06 [0.95, 1.19]  1.07 [0.92, 1.25] | Daily energy intake, alcohol consumption, level of education, family history of diabetes, physical activity, hypertension,  hypercholesterolaemia, smoking status, tertile groups of remaining fatty acid groups and BMI  (age as time-scale in cox regression model) |
|  |  |  |  |  |  | MUFA | <28.7 g/d (REF)  28.7-33.3 g/d  ≥33.3 g/d | 1.00  1.01 [0.91, 1.12]  1.06 [0.95, 1.19] |  |
|  |  |  |  |  |  | PUFA | <12.0 g/d (REF)  12.0-15.3 g/d  ≥15.3 g/d | 1.00  1.03 [0.93, 1.14]  1.06 [0.96, 1.17] |  |
|  |  |  |  |  |  | Total omega-3 | <1.3 g/d (REF)  1.3-1.6 g/d  ≥1.6 g/d | 1.00  1.10 [0.99, 1.22]  1.26 [1.13, 1.41] |  |
|  |  |  |  |  |  | EPA | <0.09 g/d (REF)  0.09-0.20 g/d  ≥0.20 g/d | 1.00  0.88 [0.73, 1.06]  0.88 [0.67, 1.15] |  |
|  |  |  |  |  |  | DHA | <0.19 g/d (REF)  0.19-0.38 g/d  ≥0.38 g/d | 1.00  1.15 [0.95, 1.38]  1.11 [0.85, 1.44] |  |
|  |  |  |  |  |  | ALA | <0.90 g/d (REF)  0.90-1.14 g/d  ≥1.14 g/d | 1.00  1.00 [0.90, 1.12]  1.03 [0.92, 1.15] |  |
|  |  |  |  |  |  | Total omega-6 | <10.5 g/d (REF)  10.5-13.7 g/d  ≥13.7 g/d | 1.00  1.01 [0.91, 1.12]  1.00 [0.90, 1.10] |  |
|  |  |  |  |  |  | LA | <10.3 g/d (REF)  10.3-13.5 g/d  ≥13.5 g/d | 1.00  0.98 [0.89, 1.08]  0.97 [0.87, 1.07] |  |
|  |  |  |  |  |  | AA | <0.19 g/d (REF)  0.19-0.25 g/d  ≥0.25 g/d | 1.00  1.11 [0.99, 1.24]  1.49 [1.33, 1.66] |  |
|  |  |  |  |  |  | TFA | <1.4 g/d (REF)  1.4-1.7 g/d  ≥1.7 g/d | 1.00  0.90 [0.80, 1.00]  1.02 [0.89, 1.17] |  |
| Ericson, 2015, Sweden, Malmö Diet and Cancer  Cohort (MDC)  SRef (6) | 14 years | Interview-based, modified diet history  method (validated):  - 7-d menu book  - 168-item FFQ  - a 45-min  interview | 26,930, m/w, 45-74 years | 2860 | via at least one of 7 registries or at new screenings or examinations during follow-up  information on date of diagnosis was used from 2 registries (the regional Diabetes 2000  registry of Scania and the Swedish National Diabetes Registry) that required a physician diagnosis  according to established diagnosis criteria: fasting plasma glucose  concentration ≥7.0 mmol/L or fasting whole blood concentration  ≥6.1 mmol/L, measured at 2 different occasions. | Total fat | 31 E% (REF)  35 E%  38 E%  41 E%  46 E% | 1.00  1.00 [0.88, 1.12]  0.95 [0.85, 1.07]  0.93 [0.83, 1.05]  0.96 [0.85, 1.08] | Age, sex, method version, season, total energy intake, leisure-time physical activity, smoking, alcohol intake, education and BMI |
|  |  |  |  |  |  | SFA | 12 E% (REF)  14 E%  16 E%  18 E%  22 E% | 1.00  1.07 [0.96, 1.19]  0.91 [0.81, 1.02]  0.93 [0.82, 1.04]  0.91 [0.81, 1.02] |  |
|  |  |  |  |  |  | MUFA | 11 E% (REF)  12 E%  13 E%  14 E%  16 E% | 1.00  0.99 [0.88, 1.12]  0.98 [0.87, 1.10]  1.03 [0.91, 1.16]  1.01 [0.89, 1.13] |  |
|  |  |  |  |  |  | PUFA | 4 E% (REF)  5 E%  6 E%  7 E%  8 E% | 1.00  1.08 [0.96, 1.22]  1.04 [0.92, 1.17]  1.08 [0.96, 1.22]  1.07 [0.95, 1.20] |  |
|  |  |  |  |  |  | Total omega-3 | 0.7 E% (REF)  0.8 E%  0.9 E%  1.1 E%  1.4 E% | 1.00  0.90 [0.80, 1.02]  0.91 [0.81, 1.02]  0.93 [0.83, 1.05]  1.00 [0.89, 1.12] |  |
|  |  |  |  |  |  | Long-chain omega-3 | 0.07 E% (REF)  0.12 E%  0.19 E%  0.29 E%  0.52 E% | 1.00  1.01 [0.90, 1.14] 0.99 [0.88, 1.12]  0.92 [0.81, 1.04]  1.07 [0.94, 1.20] |  |
|  |  |  |  |  |  | ALA | 0.5 E% (REF)  0.6 E%  0.7 E%  0.8 E%  1.0 E% | 1.00  **0.85 [0.76, 0.95]**  0.94 [0.84, 1.05]  **0.85 [0.76, 0.95]**  0.94 [0.83, 1.05] |  |
|  |  |  |  |  |  | Total omega-6 | 3.2 E% (REF)  4.0 E%  4.7 E%  5.5 E%  6.8 E% | 1.00  1.13 [1.00, 1.28]  1.07 [0.95, 1.21]  1.11 [0.98, 1.25]  1.09 [0.97, 1.23] |  |
|  |  |  |  |  |  | Omega-3:omega-6 - ratio | 0.14 (REF)  0.17  0.19  0.23  0.30 | 1.00  0.90 [0.80, 1.01]  1.00 [0.90, 1.13]  0.98 [0.87, 1.10]  0.91 [0.81, 1.03] |  |
|  |  |  |  |  |  | ALA:LA ratio | 0.11 (REF)  0.14  0.15  0.17  0.21 | 1.00  0.91 [0.81, 1.02]  0.93 [0.83, 1.04]  1.03 [0.93, 1.16]  0.86 [0.76, 0.97] |  |
| Guasch-Ferre, 2017, Spain, PREDIMED  SRef (7) | 4.3 years | Validated  semiquantitative FFQ, completed in a face-to-  face interview by trained dieticians | 3349, m/w, 55-80 years | 266 | T2D incidence  diagnosed according to ADA criteria | Total fat | 30.55 En% (REF)  36.23 En%  40.43 En%  45.53 En% | 1.00  1.06 [0.69, 1.61]  1.03 [0.67, 1.57]  1.69 [1.12, 2.54] | Age, sex, intervention group, BMI, smoking status, educational status, leisure-time physical activity, baseline hypertension or use of antihypertensive medication, total energy intake, alcohol intake, quartiles of fiber, protein intake, dietary cholesterol, specific types of fat, hypercholesterolemia or use of lipid-lowering drugs and fasting plasma glucose at baseline |
|  |  |  |  |  |  | Animal fat | 9.37 En% (REF)  12.48 En%  15.01 En%  18.57 En% | 1.00  1.22 [0.79, 1.86]  1.22 [0.80, 1.87]  1.24 [0.78, 1.98] |  |
|  |  |  |  |  |  | Vegetable fat | 17.07 En% (REF)  21.97 En%  26.31 En%  31.48 En% | 1.00  0.97 [0.64, 1.45]  1.27 [0.86, 1.87]  1.62 [1.07, 2.47] |  |
|  |  |  |  |  |  | MUFA | 14.12 En% (REF)  17.54 En%  20.31 En%  23.90 En% | 1.00  0.83 [0.55, 1.28]  0.87 [0.57, 1.33]  1.10 [0.71, 1.71] |  |
|  |  |  |  |  |  | PUFA | 4.14 En% (REF)  5.20 En%  6.23 En%  8.28 En% | 1.00  1.25 [0.81, 1.91]  1.32 [0.85, 2.05]  1.56 [1.03, 2.35] |  |
|  |  |  |  |  |  | Marine omega-3 | 0.14 En% (REF)  0.23 En%  0.32 En%  0.57 En% | 1.00  1.28 [0.87, 1.88]  1.06 [0.69, 1.61]  1.10 [0.71, 1.72] |  |
|  |  |  |  |  |  | Nonmarine omega-3 | 0.35 En% (REF)  0.44 En%  0.55 En%  0.80 En% | 1.00  1.20 [0.78, 1.84]  1.20 [0.75, 1.93]  1.19 [0.72, 1.97] |  |
|  |  |  |  |  |  | LA | 3.24 En% (REF)  4.21 En%  5.20 En%  7.11 En% | 1.00  1.46 [0.95, 2.25]  1.47 [0.91, 2.37]  1.59 [0.96, 2.63] |  |
|  |  |  |  |  |  | SFA | 7.29 En% (REF)  8.92 En%  10.31 En%  12.21 En% | 1.00  1.31 [0.84, 2.06]  1.21 [0.74, 1.98]  1.16 [0.67, 1.99] |  |
|  |  |  |  |  |  | TFA | 0.08 En% (REF)  0.15 En%  0.23 En%  0.38 En% | 1.00  1.59 [1.03, 2.45]  1.22 [0.77, 1.95]  1.26 [0.76, 2.11] |  |
| Ha, 2019, South Korea, Korean Genome and Epidemiology  Study (KoGES)  SRef (8) | 11.5 years | Validated 103-item semi-quantitative FFQ | 5595, m/w, 40-69 years | 1010 | According to WHO guidelines:  (1) fasting blood glucose level ≥126 mg/dl, (2)  2-h blood glucose level ≥200 mg/dl, (3) physician’s diagnosis  of diabetes, and (4) treatment with oral hyperglycemic medication  or insulin | Total fat | 10.2 En%  13.5 En%  16.3 En%  20.2 En% (REF) | 1.40 (1.00, 1.96)  1.22 (0.92, 1.61)  0.96 (0.74, 1.25)  1.00 | Alcohol consumption, BMI, education level, household income level, marital status, smoking status, parental history of diabetes, physical activity, residence, protein intake (% of total energy), total energy intake, fasting blood glucose at baseline |
| Kaushik, 2009, USA, Nurses’ Health Study (NHS)  SRef (9) | 29 years | Validated semi-quantitative FFQ, 120 items | 61,031, w, 30-55 years | 4159 | Self-report, validated according to the National Diabetes Data Group criteria | Long-chain omega-3 | 0.06 g/d (REF)  0.12 g/d  0.18 g/d  0.27 g/d  0.49 g/d | 1.00  1.00 (0.91, 1.11)  1.12 (1.02, 1.24)  1.17 (1.05, 1.29)  1.23 (1.11, 1.37) | Age, smoking, alcohol consumption, physical activity, family history of diabetes, BMI, intakes of SFA, TFA, ALA, LA, caffeine, cereal fiber, glycemic index, calories, menopausal status and postmenopausal hormone use |
| Kaushik, 2009, USA, Nurses’ Health Study II (NHS II)  SRef (9) | 15 years | Validated semi-quantitative FFQ, 120 items | 61,669, w, 26-46 years | 2728 | Self-report, validated according to the National Diabetes Data Group criteria | Long-chain omega-3 | 0.06 g/d (REF)  0.10 g/d  0.15 g/d  0.22 g/d  0.36 g/d | 1.00  1.04 (0.92, 1.17)  1.08 (0.95, 1.22)  1.15 (1.02, 1.30)  1.25 (1.10, 1.42) | Age, smoking, alcohol consumption, physical activity, family history of diabetes, BMI, intakes of SFA, TFA, ALA, LA, caffeine, cereal fiber, glycemic index, calories, hormone replacement therapy and contraceptive use |
| Kaushik, 2009, USA, Health Professional Follow-up Study (HPFS)  SRef (9) | 18 years | Validated semi-quantitative FFQ, 120 items | 42,504, m, 39-78 years | 2493 | Self-report, validated according to the National Diabetes Data Group criteria | Long-chain omega-3 | 0.09 g/d (REF)  0.18 g/d  0.28 g/d  0.39 g/d  0.62 g/d | 1.00  1.00 (0.88, 1.13)  0.99 (0.87, 1.12)  1.11 (0.98, 1.26)  1.12 (0.98, 1.28) | Age, smoking, alcohol consumption, physical activity, family history of diabetes, BMI, intakes of SFA, TFA, ALA, LA, caffeine, cereal fiber, glycemic index and calories |
| Kröger, 2011, Germany, EPIC-Potsdam  SRef (10) | 7 years | Self-administered validated FFQ | 2714, m/w, 50 years | 670 | The prevalence of diabetes at baseline was evaluated by  a physician who used information on self-reported medical diagnoses,  medication records, and dieting behavior. Uncertainties  regarding a proper diagnosis at baseline were clarified with the  participant or treating physician. | SFA | 36.1 E% fat (REF)  39.1 E% fat  41.1 E% fat  43.5 E% fat  47.0 E% fat | 1.00  0.71 (0.51, 1.00)  0.86 (0.63, 1.18)  0.73 (0.52, 1.02)  0.71 (0.50, 0.99) | Age, sex, BMI, waist circumference, cycling, sports activity, education, smoking status, alcohol intake,  occupational activity, coffee intake (energy adjusted), fiber intake (energy adjusted), total fat intake, and total energy intake. |
|  |  |  |  |  |  | MUFA | 31.8 E% fat (REF)  33.0 E% fat  33.8 E% fat  34.7 E% fat  36.1 E% fat | 1.00  0.80 (0.57, 1.13)  1.05 (0.75, 1.46)  0.79 (0.56, 1.12)  0.98 (0.71, 1.35) |  |
|  |  |  |  |  |  | PUFA | 11.6 E% fat (REF)  14.8 E% fat  17.5 E% fat  20.2 E% fat  24.5 E% fat | 1.00  1.01 (0.71, 1.43)  1.30 (0.91, 1.86)  1.21 (0.86, 1.72)  1.26 (0.89, 1.77) |  |
|  |  |  |  |  |  | Long-chain omega-3 | 0.04 E% fat (REF)  0.16 E% fat  0.23 E% fat  0.32 E% fat  0.59 E% fat | 1.00  1.01 (0.71, 1.46)  0.82 (0.58, 1.18)  0.97 (0.69, 1.36)  1.29 (0.95, 1.75) |  |
|  |  |  |  |  |  | ALA | 1.4 E% fat (REF)  1.7 E% fat  1.9 E% fat  2.1 E% fat  2.6 E% fat | 1.00  1.13 (0.80, 1.59)  1.27 (0.90, 1.80)  1.31 (0.93, 1.85)  1.13 (0.80, 1.59) |  |
|  |  |  |  |  |  | LA | 9.0 E% fat (REF)  12.1 E% fat  14.8 E% fat  17.4 E% fat  21.8 E% fat | 1.00  0.90 (0.63, 1.28)  1.14 (0.80, 1.63)  1.08 (0.76, 1.54)  1.11 (0.79, 1.56) |  |
| Lindstrom, 2006, Finland, Finnish DPS  SRef (11) | 4.1 years | 3-day food record at baseline and before every annual study visit | 500, m/w, 55 years | 114 | Diabetes was  defined according to the WHO 1985 criteria | Total fat | <30 E% (REF)  30-33.16 E%  33.16-36.86 E%  >36.86 E% | 1.00  1.07 [0.53, 2.15]  1.40 [0.74, 2.64]  2.14 [1.16, 3.92] | Intervention assignment, sex, age, baseline weight, baseline 2-h glucose, physical activity at baseline and during follow-up period, baseline intake of explanatory nutrient, weight change |
|  |  |  |  |  |  | SFA | <12.14 E% (REF)  12.14-14.4 E%  14.4-16.63 E%  >16.63 E% | 1.00  1.15 [0.58, 2.29]  1.99 [1.09, 3.64]  1.73 [0.89, 3.38] |  |
| Ma, 2015, USA, Cardiovascular health study (CHS)  SRef (12) | 16 years | Validated picture-sort FFQ in 1989–1990 and a FFQ (1995–1996 examination) | 4221, m/w, ≥65 years | 297 | 1) the new use of insulin or oral hypoglycemic agents, 2) a  fasting glucose concentration ≥7 mmol/L (126 mg/dL), or 3) a  nonfasting glucose concentration ≥11.1 mmol/L (200 mg/dL). | Myristic acid | 0.53 E% (REF)  0.66 E%  0.79 E%  0.92 E%  1.1 E% | 1.00  0.97 [0.72, 1.32]  0.87 [0.63, 1.21]  1.01 [0.71, 1.43]  1.00 [0.66, 1.52] | Age, sex, race, education, clinic, smoking status, alcohol consumption, physical activity, prevalence of ischemic heart disease, hypertension at baseline, BMI, and consumption of saturated fat (E%) and monounsaturated fat  (E%) with each fatty acid excluded, polyunsaturated fat (E%), trans-fat (E%), protein (E%), and total energy |
|  |  |  |  |  |  | Palmitic acid | 4.2 E% (REF)  5.1 E%  5.9 E%  6.6 E%  7.5 E% | 1.00  0.99 [0.73, 1.36]  1.01 [0.72, 1.43]  1.02 [0.69, 1.51]  1.14 [0.72, 1.79] |  |
|  |  |  |  |  |  | Stearic acid | 1.7 E% (REF)  2.2 E%  2.6 E%  2.9 E%  3.4 E% | 1.00  1.06 [0.77, 1.46]  1.06 [0.74, 1.50]  0.99 [0.66, 1.48]  1.00 [0.62, 1.62] |  |
|  |  |  |  |  |  | Palmitoleic acid | 0.38 E% (REF)  0.51 E%  0.63 E%  0.75 E%  0.92 E% | 1.00  1.05 [0.76, 1.45]  1.37 [0.99, 1.89]  1.08 [0.75, 1.56]  1.13 [0.75, 1.71] |  |
|  |  |  |  |  |  | Oleic acid | 7.3 E% (REF)  9.2 E%  10.4 E%  11.7 E%  13.3 E% | 1.00  1.01 [0.73, 1.41]  0.93 [0.66, 1.32]  1.02 [0.71. 1.47]  0.99 [0.66, 1.49] |  |
| Meyer, 2001, USA, Iowa Women’s Health Study (IWHS)  SRef (13) | 11 years | Validated 127-item FFQ | 35,988, w, 55-69 years | 1890 | Self-report, with validation of 85 cohort participants in 1988 | Total fat | 55.7 g/d (REF)  56.1 g/d  60.1 g/d  66.8 g/d  86.6 g/d | 1.00  1.00 (0.85, 1.17)  0.95 (0.81, 1.11)  0.93 (0.79, 1.10)  0.89 (0.75, 1.05) | Age, total energy, WHR, BMI, physical activity, cigarette smoking, alcohol consumption, education, marital status, residential area, hormone replacement therapy, dietary magnesium and cereal fiber |
|  |  |  |  |  |  | SFA | 19.3 g/d (REF)  19.2 g/d  20.4 g/d  23.2 g/d  31.8 g/d | 1.00  1.05 (0.89, 1.24)  1.06 (0.90, 1.25)  1.10 (0.94, 1.29)  1.00 (0.85, 1.18) |  |
|  |  |  |  |  |  | PUFA | 8.9 g/d (REF)  9.2 g/d  10.4 g/d  12.2 g/d  16.6 g/d | 1.00  0.94 (0.81, 1.08)  0.91 (0.78, 1.06)  0.85 (0.73, 0.99)  0.88 (0.76, 1.02) |  |
|  |  |  |  |  |  | MUFA | 20.4 g/d (REF)  20.9 g/d  22.7 g/d  25.7 g/d  33.8 g/d | 1.00  0.99 (0.84, 1.16)  1.01 (0.86, 1.19)  0.90 (0.76, 1.06)  0.96 (0.82, 1.13) |  |
|  |  |  |  |  |  | Long-chain omega-3 | 0.03 g/d (REF)  0.09 g/d  0.13 g/d  0.20 g/d  0.39 g/d | 1.00  0.98 (0.84, 1.14)  1.01 (0.87, 1.18)  0.99 (0.85, 1.15)  1.20 (1.03, 1.39) |  |
|  |  |  |  |  |  | TFA | 2.2 g/d (REF)  2.4 g/d  2.8 g/d  3.5 g/d  5.2 g/d | 1.00  0.99 (0.85, 1.15)  0.90 (0.77, 1.05)  0.82 (0.70, 0.97)  0.83 (0.70, 0.97) |  |
|  |  |  |  |  |  | Animal fat | 29.1 g/d (REF)  29.8 g/d  33.7 g/d  40.4 g/d  56.8 g/d | 1.00  1.06 (0.90, 1.25)  1.04 (0.89, 1.23)  1.12 (0.95, 1.31)  1.09 (0.93, 1.28) |  |
|  |  |  |  |  |  | Vegetable fat | 18.6 g/d (REF)  20.2 g/d  23.7 g/d  29.2 g/d  41.7 g/d | 1.00  0.86 (0.76, 1.03)  0.85 (0.73, 0.99)  0.81 (0.70, 0.95)  0.79 (0.68, 0.92) |  |
| Salmeron, 1997, USA, Health Professional’s Follow-Up Study (HPFS)  SRef (14) | 6 years | Validated semi-quantitative 131-item FFQ | 42,759, m, 40-75 years | 523 | According the criteria of NIDDM  proposed by the National Diabetes Data Group (1979) and the World Health Organization (1985) | Vegetable fat | 18.5 g/d (REF)  24.5 g/d  29.1 g/d  34.0 g/d  42.6 g/d | 1.00  0.95 (0.73, 1.24)  0.95 (0.72, 1.25)  0.99 (0.75, 1.30)  0.83 (0.62, 1.11) | Age, BMI, alcohol intake, smoking status, physical activity and family history of diabetes |
|  |  |  |  |  |  | Animal fat | 26.5 g/d (REF)  34.5 g/d  49.5 g/d  47.0 g/d  57.3 g/d | 1.00  0.89 (0.64, 1.25)  1.19 (0.87, 1.62)  1.19 (0.88, 1.62)  1.11 (0.82, 1.50) |  |
|  |  |  |  |  |  | SFA | 16.7 g/d (REF)  21.3 g/d  24.3 g/d  27.3 g/d  32.1 g/d | 1.00  1.20 (0.87, 1.65)  1.31 (0.96, 1.78)  1.17 (0.85, 1.59)  1.03 (0.75, 1.41) |  |
|  |  |  |  |  |  | PUFA | 9.2 g/d (REF) 11.3 g/d  12.8 g/d  14.5 g/d  17.4 g/d | 1.00  1.00 (0.76, 1.34)  1.00 (0.76, 1.34)  1.16 (0.89, 1.54)  1.01 (0.77, 1.35) |  |
|  |  |  |  |  |  | MUFA | 19.5 g/d (REF)  24.2 g/d  27.3 g/d  30.2 g/d  36.6 g/d | 1.00  1.04 (0.76, 1.42)  1.26 (0.93, 1.69)  1.16 (0.85, 1.56)  1.01 (0.74, 1.37) |  |
| Salmeron, 2001, USA, Nurses’ Health Study (NHS)  SRef (15) | 14 years | Validated semi-quantitative FFQ including 61-items (1980) and 116-136 items (1840 and on) | 84,204, w, 30-55 years | 2507 | According the criteria of NIDDM  proposed by the National Diabetes Data Group (1979) and the World Health Organization (1985) | Total fat | 28.9 E% (REF)  33.9 E%  37.2 E%  40.6 E%  46.1 E% | 1.00  0.87 (0.77, 1.00)  1.01 (0.88, 1.15)  0.97 (0.85, 1.10)  0.97 (0.85, 1.11) | Age, time period, BMI, cigarette smoking, parental history of diabetes, alcohol consumption, physical activity, percentage of energy from protein, total energy intake and dietary cholesterol |
|  |  |  |  |  |  | Animal fat | 17.3 E% (REF)  21.6 E%  25.0 E%  29.2 E%  36.4 E% | 1.00  0.88 (0.76, 1.02)  1.00 (0.86, 1.15)  1.02 (0.88, 1.19)  0.97 (0.82, 1.15) |  |
|  |  |  |  |  |  | Vegetable fat | 5.3 E% (REF)  8.7 E%  11.1 E%  13.5 E%  17.2 E% | 1.00  0.85 (0.75, 0.96)  0.67 (0.59, 0.77)  0.65 (0.56, 0.76)  0.60 (0.51, 0.71) |  |
|  |  |  |  |  |  | SFA | 10.7 E% (REF)  12.8 E%  14.3 E%  16.0 E%  18.8 E% | 1.00  0.97 (0.83, 1.12)  0.96 (0.81, 1.14)  1.03 (0.86, 1.24)  0.99 (0.80, 1.21) |  |
|  |  |  |  |  |  | MUFA | 10.9 E% (REF)  13.1 E%  14.6 E%  16.3 E%  19.3 E% | 1.00  1.07 (0.91, 1.25)  1.05 (0.88, 1.26)  1.02 (0.83, 1.25)  1.06 (0.84, 1.33) |  |
|  |  |  |  |  |  | PUFA | 2.9 E% (REF)  3.4 E%  4.1 E%  4.8 E%  6.2 E% | 1.00  0.86 (0.76, 0.97)  0.77 (0.67, 0.88)  0.75 (0.65, 0.86)  0.75 (0.65, 0.88) |  |
|  |  |  |  |  |  | TFA | 1.3 E% (REF)  1.7 E%  2.0 E%  2.4 E%  2.9 E% | 1.00  1.12 (0.97, 1.29)  1.18 (1.02, 1.37)  1.14 (0.97, 1.34)  1.31 (1.10, 1.56) |  |
| Song, 2004, USA, Women’s Health Study (WHS)  SRef (16) | 8.8 years | Validated, semi-quantitative FFQ | 37,309, w, 53.5 – 54.6 years | 1558 | Self-report, validation in subgroups via blood samples and telephone interviews according to the ADA criteria | SFA | 13.8 g/d (REF)  17.1 g/d  19.4 g/d  21.8 g/d  25.8 g/d | 1.00  0.95 (0.76, 1.18)  0.94 (0.73, 1.21)  0.85 (0.64, 1.13)  0.89 (0.66, 1.21) | Age, BMI, total energy intake, smoking, exercise, alcohol use, family history of diabetes, fiber intake, glycemic load, magnesium and total fat |
|  |  |  |  |  |  | MUFA | 15.1 g/d (REF)  18.9 g/d  21.4 g/d  24.0 g/d  27.9 g/d | 1.00  1.01 (0.77, 1.32)  1.11 (0.80, 1.55)  1.17 (0.81, 1.70)  1.27 (0.85, 1.91) |  |
|  |  |  |  |  |  | Omega-3 | 0.95 g/d (REF)  1.17 g/d  1.34 g/d  1.54 g/d  1.88 g/d | 1.00  1.09 (0.92, 1.29)  1.06 (0.89, 1.25)  1.13 (0.96, 1.34)  1.10 (0.93, 1.30) |  |
|  |  |  |  |  |  | Omega-6 | 7.35 g/d (REF)  9.12 g/d  10.5 g/d  12.0 g/d  14.5 g/d | 1.00  1.06 (0.89, 1.26)  1.09 (0.91, 1.31)  1.04 (0.86, 1.25)  0.95 (0.78, 1.16) |  |
|  |  |  |  |  |  | TFA | 1.12 g/d (REF)  1.64 g/d  2.09 g/d  2.65 g/d  3.66 g/d | 1.00  1.21 (1.00, 1.46)  1.09 (0.89, 1.33)  1.07 (0.87, 1.32)  1.03 (0.83, 1.28) |  |
|  |  |  |  |  |  | Animal fat | 19.7 g/d (REF)  26.0 g/d  30.4 g/d  35.2 g/d  43.1 g/d | 1.00  0.94 (0.78, 1.15)  1.05 (0.86, 1.29)  1.08 (0.87, 1.34)  1.13 (0.90, 1.43) |  |
|  |  |  |  |  |  | Vegetable fat | 16.9 g/d (REF)  22.0 g/d  25.9 g/d  30.2 g/d  37.3 g/d | 1.00  0.98 (0.82, 1.16)  1.08 (0.90, 1.28)  0.98 (0.81, 1.18)  0.85 (0.70, 1.03) |  |
| Van Dam, 2002, USA, Health Professionals Follow-Up Study (HPFS)  SRef (17) | 12 years | Validated semi-quantitative 131-item FFQ | 42,504, m, 40-75 years | 1321 | Self-report, validation according to WHO criteria (1985) | Total fat | 24 E% (REF)  29 E%  32 E%  35 E%  39 E% | 1.00  0.99 (0.81, 1.21)  1.14 (0.94, 1.39)  1.00 (0.82, 1.22)  0.97 (0.79, 1.18) | Age, total energy intake, time period, physical activity, cigarette smoking, alcohol consumption, hypercholesterolemia, hypertension, family history of diabetes, cereal fiber, magnesium and BMI |
|  |  |  |  |  |  | SFA | 7.6 E% (REF)  9.6 E%  11 E%  12 E%  14 E% | 1.00  1.20 (0.98, 1.46)  1.12 (0.92, 1.38)  1.22 (1.00, 1.49)  0.97 (0.79, 1.20) |  |
|  |  |  |  |  |  | Oleic acid | 8 E% (REF)  10 E%  11 E%  12 E%  14 E% | 1.00  1.02 (0.84, 1.24)  1.09 (0.90, 1.32)  1.09 (0.90, 1.33)  0.93 (0.76, 1.14) |  |
|  |  |  |  |  |  | LA | 3.5 E% (REF)  4.4 E%  4.9 E%  5.6 E%  6.8 E% | 1.00  0.99 (0.83, 1.18)  1.03 (0.86, 1.23)  1.06 (0.89, 1.26)  0.89 (0.74, 1.06) |  |
|  |  |  |  |  |  | ALA | 321 mg/d (REF)  396 mg/d  458 mg/d  533 mg/d  671 mg/d | 1.00  1.03 (0.86, 1.23)  1.10 (0.92, 1.31)  1.00 (0.84, 1.20)  0.93 (0.78, 1.11) |  |
|  |  |  |  |  |  | Long-chain omega.3 | 80 mg/d (REF)  155 mg/d  250 mg/d  350 mg/d  570 mg/d | 1.00  1.01 (0.85, 1.19)  0.95 (0.79, 1.13)  1.05 (0.88, 1.25)  1.01 (0.84, 1.21) |  |
|  |  |  |  |  |  | TFA | 0.7 E% (REF)  1.0 E%  1.3 E%  1.5 E%  2.0 E% | 1.00  0.95 (0.79, 1.15)  0.93 (0.77, 1.12)  0.91 (0.75, 1.11)  0.90 (0.74, 1.10) |  |
| Van Woudenbergh, 2009, Netherlands, Rotterdam Study  SRef (18) | 12.4 years | Validated semi-quantitative 170-item FFQ | 4472, m/w, 67.2 years | 463 | Defined according to WHO (1999) and ADA criteria (1997) | Long-chain omega-3 | 23.8 mg/d (REF)  89.4 mg/d  236.8 mg/d | 1.00  1.06 (0.84, 1.34)  1.05 (0.80, 1.38) | Age, sex, smoking, education level, intake of energy, alcohol, TFA, fiber, selenium, Vitamin D and cholesterol |
| Villegas, 2011, China, Shangai Women’s Health Study (SWHS)  SRef (19) | 8.9 years | Validated FFQ | 64,193, w, 40-70 years | 2262 | Self-report and confirmation according to ADA criteria | Long-chain omega-3 | 0.02 g/d (REF)  0.04 g/d  0.07 g/d  0.11 g/d  0.20 g/d | 1.00  0.90 (0.80, 1.00)  0.84 (0.75, 0.94)  0.87 (0.77, 0.98)  0.84 (0.74, 0.95) | Age, energy intake, WHR, BMI, smoking, alcohol consumption, physical activity, income level, education level, occupation, family history of diabetes, hypertension and dietary pattern |
| Villegas, 2011, China, Shanghai Men’s Health Study (SMHS)  SRef (19) | 4.1 years | Validated FFQ | 51,963, m, 40-74 years | 833 | Self-report and confirmation according to ADA criteria | Long-chain omega-3 | 0.02 g/d (REF)  0.04 g/d  0.07 g/d  0.11 g/d  0.20 g/d | 1.00  0.95 (0.77, 1.17)  0.86 (0.69, 1.07)  0.96 (0.77, 1.19)  0.89 (0.70, 1.12) | Age, energy intake, WHR, BMI, smoking, alcohol consumption, physical activity, income level, education level, occupation, family history of diabetes, hypertension and dietary pattern |
| Virtanen, 2014, Finland, Kuopio Ischemic Heart Disease Risk Factor Study (KHID Study)  SRef (20) | 19.3 years | 4-day food record | 2212, m, 42-60 years | 422 | Self-report, fasting plasma glucose ≥ 7.0mmol/L or 2-h oral glucose tolerance test plasma glucose ≥ 11.1 mmol/L, record linkage | Long-chain omega-3 | <0.05 g/d (REF)  0.05-0.19 g/d  0.20-0.43 g/d  >0.43 g/d | 1.00  0.80 [0.61, 1.06]  0.91 [0.70, 1.19]  0.85 [0.65, 1.12] | Age, examination year, BMI, family history of diabetes, smoking, education years, leisure-time physical activity, intake of alcohol, serum linoleic acid and energy |
|  |  |  |  |  |  | ALA | <1.02 g/d (REF)  1.02-1.41 g/d  1.42-1.83 g/d  >1.83 g/d | 1.00  0.91 [0.69, 1.20]  1.09 [0.82, 1.45]  1.06 [0.79. 1.43] |  |
| Wang, 2015, USA, Cardiovascular health study (CHS)  SRef (21) | 50,105 person-years  (follow-up in years not available) | 1989–1990: validated 99-item, picture sort FFQ, 1995-1996: validated 131-item self-administered FFQ | 4207, m/w, ≥65 years | 407 | 1) the new use of insulin or oral hypoglycemic agents, 2) a  fasting glucose concentration ≥7 mmol/L (126 mg/dL), or 3) a  nonfasting glucose concentration ≥11.1 mmol/L (200 mg/dL). | TFAs | 2.17 g/d (REF)  3.24 g/d  4.05 g/d  5.39 g/d | 1.00  1.08 [0.79, 1.48]  1.13 [0.81, 1.57]  1.31 [0.92, 1.86] | Age, sex, race, education, enrolment site, smoking site, alcohol consumption, prevalence of physical activity, BMI, waist circumference, CVD, hypertension at baseline, total energy intake, dietary score that comprised  consumption of whole grains, fish, fruits and vegetables, nuts and seeds, red and processed meat, sugar-sweetened beverages, and fried potatoes |
| Zheng, 2018, China, Guangzhou Nutrition and Health Study (GNHS)  SRef (22) | 5.6 years | Validated FFQ | 2671, m,w, 40-75 years | 213 | Defined according to ADA criteria | Long-chain omega-3 | 0.021 g/d (REF)  0.042 g/d  0.068 g/d  0.12 g/d | 1.00  0.75 [0.50, 1.14]  0.87 [0.59, 1.29]  0.78 [0.52, 1.19] | Age, sex, BMI, WHR, physical activity, education, alcohol, smoking, household income, family history of diabetes, total energy intake, intake of dairy products, red and processed meat, fruits and vegetables, fasting blood glucose and erythrocyte total n-6 PUFA |
|  |  |  |  |  |  | EPA | 0.008 g/d (REF)  0.016 g/d  0.025 g/d  0.042 g/d | 1.00  0.77 [0.51, 1.15]  0.85 [0.57, 1.27]  0.76 [0.50, 1.16] |  |
|  |  |  |  |  |  | DHA | 0.011 g/d (REF)  0.024 g/d  0.039 g/d  0.067 g/d | 1.00  0.77 [0.51, 1.17]  0.87 [0.59, 1.30]  0.74 [0.49, 1.13] |  |
|  |  |  |  |  |  | ALA | 0.49 g/d (REF)  0.66 g/d  0.84 g/d  1.19 g/d | 1.00  1.37 [0.90, 2.09]  1.11 [0.72, 1.71]  1.53 [1.01, 2.33] |  |
| Zong, 2019, USA, Nurses’ Health Study (NHS),  Health  SRef (23) | 32 years | Validated FFQ | 83,648, w, 30-55 years | 9375 | Self-report, validated according to the National Diabetes Data Group criteria | Omega-6 | 2.62 E% (REF)  3.47 E%  4.16 E%  4.95 E%  6.32 E% | 1.00  0.95 [0.89, 1.02]  1.00 [0.93, 1.07]  0.94 [0.87, 1.01]  0.97 [0.90, 1.06] | Age, ethnicity, smoking status, alcohol intake, family history of diabetes, menopausal status and postmenopausal hormone use, physical activity, multivitamin use, baseline hypertension, baseline hypercholesterolemia, updated BMI, total energy intake, intake of fruits and vegetable, total fat, *trans* fats, monounsaturated fats, other PUFAs  Age, ethnicity, smoking status, alcohol intake, family history of diabetes, menopausal status and postmenopausal hormone use, physical activity, multivitamin use, baseline hypertension, baseline hypercholesterolemia, updated BMI, total energy intake, intake of fruits and vegetable, total fat, *trans* fats, monounsaturated fats, other PUFAs |
|  |  |  |  |  |  | Linoleic acid | 2.54 E% (REF)  3.39 E%  4.07 E%  4.86 E%  6.23 E% | 1.00  0.96 [0.89, 1.02]  0.99 [0.93, 1.07]  0.96 [0.89, 1.03]  0.98 [0.91, 1.06] |  |
| Zong, 2019, USA, Nurses’ Health Study II (NHS II)  SRef (23) | 32 years | Validated FFQ | 88,610, w, 25-44 years | 5460 | Self-report, validated according to the National Diabetes Data Group criteria | Omega-6 | 3.41 E% (REF)  4.17 E%  4.76 E%  5.43 E%  6.60 E% | 1.00  0.93 [0.85, 1.02]  0.91 [0.82, 1.00]  0.94 [0.85, 1.04]  0.91 [0.80, 1.02] |  |
|  |  |  |  |  |  | Linoleic acid | 3.33 E% (REF)  4.08 E%  4.68 E%  5.35 E%  6.51 E% | 1.00  0.95 [0.87, 1.04]  0.91 [0.82, 1.00]  0.94 [0.85, 1.04]  0.93 [0.82, 1.05] |  |
| Zong, 2019, USA, Professionals Follow-up Study (HPFS)  SRef (23) | 26 years | Validated FFQ | 41,771, m, 40.75 years | 3607 | Self-report, validated according to the National Diabetes Data Group criteria | Omega-6 | 3.53 E%  4.43 E%  5.13 E%  5.91 E%  7.24 E% | 1.00  0.86 [0.77, 0.96]  0.90 [0.80, 1.01]  0.82 [0.73, 0.92]  0.74 [0.65, 0.85] | Age, ethnicity, smoking status, alcohol intake, family history of diabetes, physical activity, multivitamin use, baseline hypertension, baseline hypercholesterolemia, updated BMI, total energy intake, intake of fruits and vegetable, total fat, *trans* fats, monounsaturated fats, other PUFAs |
|  |  |  |  |  |  | Linoleic acid | 3.45 E%  4.35 E%  5.05 E%  5.83 E%  7.16 E% | 1.00  0.87 [0.78, 0.97]  0.88 [0.79, 0.99]  0.83 [0.74, 0.94]  0.77 [0.67, 0.88] |  |

FFQ: food frequency questionnaire, BMI: body mass index, MUFAs: monounsaturated fatty acids, PUFAs: polyunsaturated fatty acids, ALA: alpha linolenic acid, EPA: eicosapentaenoic acid, DHA: docosahexaenoic acid, SFAs: saturated fatty acids, TFAs: *trans*-fatty acids, E%: percent of total energy intake, E% fat: energy percent of total fat intake, ADA: American Diabetes Association, WHR: waist-to-hip ratio

**References**

1. Alhazmi A, Stojanovski E, McEvoy M, Garg ML. Macronutrient intake and type 2 diabetes risk in middle-aged Australian women. Results from the Australian Longitudinal Study on Women's Health. Public Health Nutr. 2014;17(7):1587-94.

2. Brostow DP, Odegaard AO, Koh WP, Duval S, Gross MD, Yuan JM, et al. Omega-3 fatty acids and incident type 2 diabetes: the Singapore Chinese Health Study. Am J Clin Nutr. 2011;94(2):520-6.

3. Djousse L, Gaziano J, Buring JE, Lee IM. Dietary omega-3 fatty acids and fish consumption and risk of type 2 diabetes. American Journal of Clinical Nutrition. 2011;93(1):143-50.

4. Djousse L, Biggs ML, Lemaitre RN, King IB, Song X, Ix JH, et al. Plasma omega-3 fatty acids and incident diabetes in older adults. Am J Clin Nutr. 2011;94(2):527-33.

5. Dow C, Mangin M, Balkau B, Affret A, Boutron-Ruault MC, Clavel-Chapelon F, et al. Fatty acid consumption and incident type 2 diabetes: an 18-year follow-up in the female E3N (Etude Epidemiologique aupres des femmes de la Mutuelle Generale de l'Education Nationale) prospective cohort study. Br J Nutr. 2016:1-9.

6. Ericson U, Hellstrand S, Brunkwall L, Schulz CA, Sonestedt E, Wallstrom P, et al. Food sources of fat may clarify the inconsistent role of dietary fat intake for incidence of type 2 diabetes. Am J Clin Nutr. 2015;101(5):1065-80.

7. Guasch-Ferre M, Becerra-Tomas N, Ruiz-Canela M, Corella D, Schroeder H, Estruch R, et al. Total and subtypes of dietary fat intake and risk of type 2 diabetes mellitus in the Prevencion con Dieta Mediterranea (PREDIMED) study. American Journal of Clinical Nutrition. 2017;105(3):723-35.

8. Ha K, Joung H, Song Y. Inadequate fat or carbohydrate intake was associated with an increased incidence of type 2 diabetes mellitus in Korean adults: A 12-year community-based prospective cohort study. Diabetes Res Clin Pract. 2019;148:254-61.

9. Kaushik M, Mozaffarian D, Spiegelman D, Manson JE, Willett WC, Hu FB. Long-chain omega-3 fatty acids, fish intake, and the risk of type 2 diabetes mellitus. Am J Clin Nutr. 2009;90(3):613-20.

10. Kroger J, Zietemann V, Enzenbach C, Weikert C, Jansen EH, Doring F, et al. Erythrocyte membrane phospholipid fatty acids, desaturase activity, and dietary fatty acids in relation to risk of type 2 diabetes in the European Prospective Investigation into Cancer and Nutrition (EPIC)-Potsdam Study. Am J Clin Nutr. 2011;93(1):127-42.

11. Lindstrom J, Peltonen M, Eriksson JG, Louheranta A, Fogelholm M, Uusitupa M, et al. High-fibre, low-fat diet predicts long-term weight loss and decreased type 2 diabetes risk: the Finnish Diabetes Prevention Study. Diabetologia. 2006;49(5):912-20.

12. Ma W, Wu JH, Wang Q, Lemaitre RN, Mukamal KJ, Djousse L, et al. Prospective association of fatty acids in the de novo lipogenesis pathway with risk of type 2 diabetes: the Cardiovascular Health Study. American Journal of Clinical Nutrition. 2015;101(1):153-63.

13. Meyer KA, Kushi LH, Jacobs DR, Jr., Folsom AR. Dietary fat and incidence of type 2 diabetes in older Iowa women. Diabetes Care. 2001;24(9):1528-35.

14. Salmeron J, Ascherio A, Rimm EB, Colditz GA, Spiegelman D, Jenkins DJ, et al. Dietary fiber, glycemic load, and risk of NIDDM in men. Diabetes care. 1997;20(4):545-50.

15. Salmeron J, Hu FB, Manson JE, Stampfer MJ, Colditz GA, Rimm EB, et al. Dietary fat intake and risk of type 2 diabetes in women. Am J Clin Nutr. 2001;73(6):1019-26.

16. Song Y, Manson JE, Buring JE, Liu S. A prospective study of red meat consumption and type 2 diabetes in middle-aged and elderly women: the women's health study. Diabetes Care. 2004;27(9):2108-15.

17. van Dam RM, Stampfer M, Willett WC, Hu FB, Rimm EB. Dietary fat and meat intake in relation to risk of type 2 diabetes in men. Diabetes Care. 2002;25(3):417-24.

18. van Woudenbergh GJ, van Ballegooijen AJ, Kuijsten A, Sijbrands EJ, van Rooij FJ, Geleijnse JM, et al. Eating fish and risk of type 2 diabetes: A population-based, prospective follow-up study. Diabetes Care. 2009;32(11):2021-6.

19. Villegas R, Xiang YB, Elasy T, Li HL, Yang G, Cai H, et al. Fish, shellfish, and long-chain n-3 fatty acid consumption and risk of incident type 2 diabetes in middle-aged Chinese men and women. Am J Clin Nutr. 2011;94(2):543-51.

20. Virtanen JK, Mursu J, Voutilainen S, Uusitupa M, Tuomainen TP. Serum omega-3 polyunsaturated fatty acids and risk of incident type 2 diabetes in men: the Kuopio Ischemic Heart Disease Risk Factor study. Diabetes Care. 2014;37(1):189-96.

21. Wang Q, Imamura F, Ma W, Wang M, Lemaitre RN, King IB, et al. Circulating and Dietary Trans Fatty Acids and Incident Type 2 Diabetes in Older Adults: The Cardiovascular Health Study. Diabetes Care. 2015;38(6):1099-107.

22. Zheng JS, Lin JS, Dong HL, Zeng FF, Li D, Song Y, et al. Association of erythrocyte n-3 polyunsaturated fatty acids with incident type 2 diabetes in a Chinese population. Clin Nutr. 2018.

23. Zong G, Liu G, Willett WC, Wanders AJ, Alssema M, Zock PL, et al. Associations Between Linoleic Acid Intake and Incident Type 2 Diabetes Among US Men and Women. Diabetes Care. 2019;42(8):1406-13.
