# Supplementary material for: Intake of dietary fats and fatty acids and the incidence of type 2 diabetes: A systematic review and dose-response meta-analysis of prospective observational studies
Source: PLoS Med. 2020 Dec 2;17(12):e1003347. doi: 10.1371/journal.pmed.1003347 (PMC7710077; doi:10.1371/journal.pmed.1003347)
Supplement: S4 Table — (DOCX) [file pmed.1003347.s012.docx]

**S4 Table:** ROBINS-I judgement for each domain and overall

| **Study** | **Bias due to confounding** | **Bias due to selection of participants** | **Bias due to exposure assessment** | **Bias due to misclassification during follow-up** | **Bias due to missing data** | **Bias due to measurement of the outcome** | **Bias due to selective reporting of the results** | **Overall judgement** |
| --- | --- | --- | --- | --- | --- | --- | --- | --- |
| **Alhazmi 2013** SRef [167] | Moderate | Moderate | Moderate | Moderate | Low | Moderate | Low | **Moderate** |
| **Brostow 2011** SRef [168] | Moderate | Low | Moderate | Moderate | Low | Moderate | Low | **Moderate** |
| **Djoussé 2011a (WHS)** SRef [169] | Moderate | Low | Moderate | Moderate | Low | Moderate | Low | **Moderate** |
| **Djoussé 2011b (CHS)** SRef [170] | Serious | Moderate | Moderate | Low | Low | Low | Low | **Serious** |
| **Dow 2016** SRef [171] | Moderate | Low | Moderate | Moderate | Moderate | Moderate | Low | **Moderate** |
| **Ericson 2015** SRef [172] | Moderate | Moderate | Moderate | Moderate | Low | Low | Low | **Moderate** |
| **Guasch-Ferre 2017** SRef [173] | Moderate | Moderate | Moderate | Low | Low | Low | Low | **Moderate** |
| **Ha 2019** SRef [174] | Moderate | Moderate | Moderate | Low | Moderate | Low | Low | **Moderate** |
| **Kaushik 2009** SRef [175] | Moderate | Low | Moderate | Low | No information | Moderate | Low | **Moderate** |
| **Kröger 2011** SRef [176] | Moderate | Low | Moderate | Moderate | Low | Low | Low | **Moderate** |
| **Lindstrom 2006** SRef [177] | Serious | Serious | Serious | Low | Low | Low | Low | **Serious** |
| **Ma 2015** SRef [178] | Moderate | Low | Moderate | Low | Low | Low | Low | **Moderate** |
| **Meyer 2001** SRef [179] | Moderate | Moderate | Moderate | Moderate | Low | Moderate | Low | **Moderate** |
| **Salmeron 1997** SRef [180] | Moderate | Moderate | Moderate | Moderate | Low | Moderate | Low | **Moderate** |
| **Salmeron 2001** SRef [181] | Moderate | Moderate | Moderate | Moderate | No information | Low | Low | **Moderate** |
| **Song 2004** SRef [182] | Moderate | Moderate | Moderate | Moderate | Low | Moderate | Low | **Moderate** |
| **Van Dam 2002** SRef [183] | Moderate | Moderate | Moderate | Low | Low | Moderate | Low | **Moderate** |
| **Van Woudenbergh 2009** SRef [184] | Moderate | Moderate | Moderate | Moderate | Moderate | Low | Low | **Moderate** |
| **Villegas 2011** SRef [185] | Moderate | Low | Moderate | Low | Low | Low | Low | **Moderate** |
| **Virtanen 2014** SRef [190] | Moderate | Low | Serious | Moderate | Low | Low | Low | **Serious** |
| **Wang 2015** SRef [187] | Moderate | Low | Moderate | Low | Low | Low | Low | **Moderate** |
| **Zheng 2018** SRef [188] | Moderate | Low | Moderate | Moderate | Moderate | Low | Low | **Moderate** |
| **Zong 2019** SRef [189] | Moderate | Moderate | Moderate | Low | Low | Moderate | Low | **Moderate** |
