# Supplementary material for: Intake of dietary fats and fatty acids and the incidence of type 2 diabetes: A systematic review and dose-response meta-analysis of prospective observational studies
Source: PLoS Med. 2020 Dec 2;17(12):e1003347. doi: 10.1371/journal.pmed.1003347 (PMC7710077; doi:10.1371/journal.pmed.1003347)
Supplement: S5 Table — (DOCX) [file pmed.1003347.s013.docx]

**S5 Table:** GRADE judgement for each domain and overall

| **Certainty assessment** | | | | | | | **No of participants / cases** | | **Effect** | **Certainty** |
| --- | --- | --- | --- | --- | --- | --- | --- | --- | --- | --- |
| **No of studies** | **Study design** | **Risk of bias** | **Inconsistency** | **Indirectness** | **Imprecision** | **Other considerations** | **Participants** | **Cases** | **Summary relative risk (95% confidence interval)** |  |
| **Total fat** | | | | | | | | | | |
| 8 | observational studies | serious ^a^ | not serious | serious ^b^ | not serious | none | 9‘089 | 207‘440 | **1.00** (0.96 to 1.05) | ⨁⨁◯◯ LOW |
| **Animal fat** | | | | | | | | | | |
| 5 | observational studies | serious ^c^ | not serious | not serious | not serious | none | 6‘744 | 203‘609 | **1.03** (1.00 to 1.06) | ⨁⨁⨁◯ MODERATE |
| **Vegetable fat** | | | | | | | | | | |
| 5 | observational studies | serious ^c^ | serious ^d^ | not serious | not serious | dose response gradient | 6‘744 | 203‘609 | **0.93** (0.82 to 1.05) | ⨁⨁⨁◯ MODERATE |
| **Saturated fatty acids** | | | | | | | | | | |
| 11 | observational studies | serious ^a^ | not serious | serious ^e^ | not serious | none | 14‘404 | 317‘423 | **0.97** (0.92 to 1.02) | ⨁⨁◯◯ LOW |
| **Monounsaturated fatty acids** | | | | | | | | | | |
| 10 | observational studies | serious ^c^ | not serious | serious ^e^ | not serious | none | 13‘492 | 317‘178 | **1.03** (0.99 to 1.08) | ⨁⨁◯◯ LOW |
| **Polyunsaturated fatty acids** | | | | | | | | | | |
| 8 | observational studies | serious ^c^ | serious ^d^ | serious ^b^ | not serious | none | 11‘637 | 275‘648 | **1.03** (0.89 to 1.20) | ⨁◯◯◯ VERY LOW |
| **Omega-6 fatty acids** | | | | | | | | | | |
| 8 | observational studies | serious ^c^ | not serious | not serious | not serious | none | 28‘033 | 401‘148 | **0.99** (0.98 to 1.00) | ⨁⨁⨁◯ MODERATE |
| **Linoleic acid** | | | | | | | | | | |
| 6 | observational studies | serious ^c^ | not serious | not serious | not serious | none | 21‘988 | 291‘426 | **0.99** (0.98 to 1.01) | ⨁⨁⨁◯ MODERATE |
| **Omega-3 fatty acids** | | | | | | | | | | |
| 5 | observational studies | serious ^c^ | serious ^d^ | serious ^b^ | not serious | none | 9‘591 | 268‘441 | **1.03** (0.98 to 1.08) | ⨁◯◯◯ VERY LOW |
| **Long-chain omega-3 fatty acids (EPA&DHA)** | | | | | | | | | | |
| 14 | observational studies | serious ^f^ | serious ^d^ | serious ^b^ | not serious | dose response gradient | 23‘761 | 445‘775 | **1.07** (1.02 to 1.13) | ⨁⨁◯◯ LOW |
| **Eicosapentaenoic acid** | | | | | | | | | | |
| 3 | observational studies | serious ^c^ | serious ^d^ | not serious | serious ^f^ | none | 5‘291 | 116‘032 | **1.11** (0.87 to 1.41) | ⨁◯◯◯ VERY LOW |
| **Docosahexaenoic acid** | | | | | | | | | | |
| 3 | observational studies | serious ^c^ | serious ^d^ | not serious | serious ^f^ | none | 5‘291 | 116‘032 | **1.16** (0.95 to 1.42) | ⨁◯◯◯ VERY LOW |
| **Alpha linolenic acid** | | | | | | | | | | |
| 9 | observational studies | serious ^f^ | not serious | serious ^b^ | not serious | none | 12‘864 | 237‘793 | **1.01** (0.97 to 1.05) | ⨁⨁◯◯ LOW |
| **Omega-6:omega-3-ratio** | | | | | | | | | | |
| 2 | observational studies | serious ^c^ | not serious | serious ^b^ | not serious | none | 2‘252 | 51‘546 | **1.00** (0.98 to 1.02) | ⨁⨁◯◯ LOW |
| ***Trans*-fatty acids** | | | | | | | | | | |
| 7 | observational studies | serious ^c^ | not serious | not serious | not serious | none | 10‘559 | 278‘895 | **1.00** (0.95 to 1.06) | ⨁⨁⨁◯ MODERATE |

#### Explanations

a. Downgraded since one study with very low weighting (total fat: 2.31% and SFAs: 1.26%) judged as serious risk of bias based on ROBINS-I was included in the meta-analysis and residual confounding cannot be ruled out; b. Source of indirectness: differences in population; c. Downgraded because residual confounding cannot be ruled out; d. Downgraded due to high heterogeneity, which could not be explained in subgroup analysis; 95% confidence intervals do not overlap; e. Source of indirectness: differences in intervention; f. Downgraded since two studies with low weighting (long-chain omega-3 fatty acids: 11.13% and ALA: 11.58%) judged as serious risk of bias based on ROBINS-I were included in the meta-analysis. Downgraded by one level, because excluding these two studies in sensitivity analyses did not change the results. Residual confounding cannot be ruled out; g. Downgraded since the 95% confidence intervals includes no effect (SRR of 1.00), but fails to exclude important harm (SRR of >1.25)
