# Supplementary material for: Intake of dietary fats and fatty acids and the incidence of type 2 diabetes: A systematic review and dose-response meta-analysis of prospective observational studies
Source: PLoS Med. 2020 Dec 2;17(12):e1003347. doi: 10.1371/journal.pmed.1003347 (PMC7710077; doi:10.1371/journal.pmed.1003347)
Supplement: S6 Table — (DOCX) [file pmed.1003347.s014.docx]

Summary RRs and 95% CIs from linear dose-response meta-analysis between total fat intake per 10 g/d with T2D incidence by subgroups^1^

|  | Cohorts, n | Summary RR (95% CI) | I^2^ (%) | P_between_^2^ |
| --- | --- | --- | --- | --- |
| All studies | 8 | 1.00 (0.96, 1.05) | 78 | - |
| Sex  Women  Men | 3  1 | 1.00 (0.96, 1.03)  0.99 (0.94, 1.05) | 41  NA | 0.920 |
| Geographic location  United States  Europe  Australia  Asia | 3  3  1  1 | 0.99 (0.96, 1.01)  1.14 (0.95, 1.36)  1.03 (0.97, 1.10)  0.81 (0.73, 0.91) | 0  88  NA  NA | 0.179  0.089  0.282 |
| Duration of follow-up, y  <10  ≥10 | 3  5 | 1.15 (0.99, 1.33)  0.97 (0.93, 1.01) | 77  68 | 0.018 |
| Number of cases  <1000  ≥1000 | 3  5 | 1.15 (0.99, 1.33)  0.97 (0.93, 1.01) | 77  68 | 0.018 |
| Outcome assessment  Self-report, validated  Blood measurements  Medical record | 3  4  1 | 0.99 (0.96, 1.03)  1.05 (0.89, 1.23)  0.98 (0.96, 1.01) | 38  89  NA | 0.707  0.725 |
| ROBINS-I  Low/moderate  Serious | 7  1 | 0.99 (0.95, 1.03)  1.50 (1.13, 1.98) | 75  NA | 0.115 |
| Adjustment for family history of diabetes  Yes  No | 3  5 | 0.95 (0.86, 1.04)  1.03 (0.97, 1.10) | 84  79 | 0.178 |

^1^ Summary RRs were calculated using a random effects model. Summary RR, Summary relative risk; 95% CI, 95% confidence interval, ^2^P_between_, P for heterogeneity between subgroups with meta-regression

Summary RRs and 95% CIs from linear dose-response meta-analysis between animal fat intake per 10 g/d with T2D incidence by subgroups^1^

|  | Cohorts, n | Summary RR (95% CI) | I^2^ (%) | P_between_^2^ |
| --- | --- | --- | --- | --- |
| All studies | 5 | 1.03 (1.00, 1.06) | 0 | - |
| Sex  Women  Men | 3  1 | 1.02 (0.99, 1.05)  1.07 (0.98, 1.17) | 0  NA | 0.768 |
| Geographic location  United States  Europe  Australia  Asia | 4  1  0  0 | 1.03 (1.00, 1.06)  1.09 (0.89, 1.32)  -  - | 0  NA  -  - | 0.557  -  - |
| Duration of follow-up, y  <10  ≥10 | 3  2 | 1.07 (1.01, 1.14)  1.02 (0.98, 1.05) | 0  0 | 0.422 |
| Number of cases  <1000  ≥1000 | 2  3 | 1.07 (0.99, 1.16)  1.02 (0.99, 1.05) | 0  0 | 0.575 |
| Outcome assessment  Self-report, validated  Blood measurements | 2  3 | 1.03 (0.99, 1.08)  1.03 (0.99, 1.07) | 0  0 | 0.492 |
| ROBINS-I  Low/moderate  Serious | 5  0 | 1.03 (1.00, 1.06)  - | 0  - | -  - |
| Adjustment for family history of diabetes  Yes  No | 3  2 | 1.03 (0.99, 1.07)  1.03 (0.98, 1.07) | 0  0 | 0.572 |

^1^ Summary RRs were calculated using a random effects model. Summary RR, Summary relative risk; 95% CI, 95% confidence interval, ^2^P_between_, P for heterogeneity between subgroups with meta-regression

Summary RRs and 95% CIs from linear dose-response meta-analysis between vegetable fat intake per 10 g/d with T2D incidence by subgroups^1^

|  | Cohorts, n | Summary RR (95% CI) | I^2^ (%) | P_between_^2^ |
| --- | --- | --- | --- | --- |
| All studies | 5 | 0.93 (0.82, 1.05) | 89 |  |
| Sex  Women  Men | 3  1 | 0.87 (0.76, 0.99)  0.94 (0.84, 1.05) | 90  NA | 0.673 |
| Geographic location  United States  Europe  Australia  Asia | 4  1  0  0 | 0.88 (0.79, 0.98)  1.16 (1.04, 1.29)  -  - | 86  NA  -  - | 0.065 |
| Duration of follow-up, y  <10  ≥10 | 3  2 | 1.00 (0.88, 1.15)  0.84 (0.69, 1.02) | 81  94 | 0.262 |
| Number of cases  <1000  ≥1000 | 2  3 | 1.05 (0.85, 1.28)  0.87 (0.76, 0.99) | 85  90 | 0.237 |
| Outcome assessment  Self-report, validated  Blood measurements | 2  3 | 0.93 (0.88, 0.97)  0.94 (0.72, 1.21) | 0.0  95 | 0.824 |
| ROBINS-I  Low/moderate  Serious | 5  0 | 0.94 (0.84, 1.05)  - | 89  - | - |
| Adjustment for family history of diabetes  Yes  No | 3  2 | 0.87 (0.75, 1.01)  1.03 (0.83, 1.29) | 88  92 | 0.325 |

^1^ Summary RRs were calculated using a random effects model. Summary RR, Summary relative risk; 95% CI, 95% confidence interval, ^2^P_between_, P for heterogeneity between subgroups with meta-regression

Summary RRs and 95% CIs from linear dose-response meta-analysis between saturated fatty acid intake per 10 g/d with T2D incidence by subgroups^1^

|  | Cohorts, n | Summary RR (95% CI) | I^2^ (%) | P_between_^2^ |
| --- | --- | --- | --- | --- |
| All studies | 11 | 0.97 (0.92, 1.02) | 34 | - |
| Sex  Women  Men | 5  1 | 0.97 (0.89, 1.04)  0.99 (0.86, 1.14) | 34  NA | 0.840 |
| Geographic location  United States  Europe  Australia  Asia | 5  5  1  0 | 0.98 (0.92, 1.05)  1.00 (0.87, 1.16)  0.86 (0.75, 0.98)  - | 0  64  NA  - | 0.231  0.201  - |
| Duration of follow-up, y  <10  ≥10 | 5  6 | 0.94 (0.77, 1.13)  0.97 (0.93, 1.00) | 57  0 | 0.269 |
| Number of cases  <1000  ≥1000 | 4  7 | 1.04 (0.83, 1.28)  0.96 (0.93, 1.00) | 65  8 | 0.664 |
| Outcome assessment  Self-report, validated  Blood measurements  Medical record | 6  4  1 | 0.95 (0.87, 1.03)  1.06 (0.92, 1.22)  0.95 (0.91, 0.99) | 40  27  NA | 0.238  0.420 |
| ROBINS-I  Low/moderate  Serious | 10  1 | 0.96 (0.92, 1.00)  1.67 (1.04, 2.67) | 9  NA | 0.123 |
| Adjustment for family history of diabetes  Yes  No | 4  7 | - 1. (0.94, 1.09)   0.95 (0.88, 1.03) | 0  47 | 0.347 |

^1^ Summary RRs were calculated using a random effects model. Summary RR, Summary relative risk; 95% CI, 95% confidence interval, ^2^P_between_, P for heterogeneity between subgroups with meta-regression

Summary RRs and 95% CIs from linear dose-response meta-analysis between monounsaturated fatty acid intake per 10 g/d with T2D incidence by subgroups^1^

|  | Cohorts, n | Summary RR (95% CI) | I^2^ (%) | P_between_^2^ |
| --- | --- | --- | --- | --- |
| All studies | 10 | 1.03 (0.99, 1.08) | 0 | - |
| Sex  Women  Men | 5  1 | 1.05 (0.97, 1.15)  1.00 (0.84, 1.18) | 36  NA | 0.833 |
| Geographic location  United States  Europe  Australia  Asia | 5  4  1  0 | 1.00 (0.94, 1.07)  1.03 (0.97, 1.10)  1.21 (1.01, 1.20)  - | 0  0  NA  - | 0.081  0.078  - |
| Duration of follow-up, y  <10  ≥10 | 5  5 | 1.09 (0.99, 1.19)  1.01 (0.96, 1.07) | 0  0 | 0.322 |
| Number of cases  <1000  ≥1000 | 5  5 | 1.07 (0.98, 1.16)  1.02 (0.96, 1.07) | 0  0 | 0.523 |
| Outcome assessment  Blood measurements  Self-report, validated Medical record | 4  5  1 | 1.02 (0.94, 1.11)  1.07 (0.97, 1.17)  1.02 (0.93, 1.11) | 0  36  NA | 0.680  0.982 |
| ROBINS-I  Low/moderate  Serious | 10  0 | 1.03 (0.99, 1.08)  - | 0  - | - |
| Adjustment for family history of diabetes  Yes  No | 4  6 | 1.05 (0.97, 1.13)  1.02 (0.97, 1.08) | 0  0 | 0.509 |

^1^ Summary RRs were calculated using a random effects model. Summary RR, Summary relative risk; 95% CI, 95% confidence interval, ^2^P_between_, P for heterogeneity between subgroups with meta-regression

Summary RRs and 95% CIs from linear dose-response meta-analysis between polyunsaturated fatty acid intake per 10 g/d with T2D incidence by subgroups^1^

|  | Cohorts, n | Summary RR (95% CI) | I^2^ (%) | P_between_^2^ |
| --- | --- | --- | --- | --- |
| All studies | 8 | 1.03 (0.89, 1.20) | 76 | - |
| Sex  Women  Men | 4  1 | 0.93 (0.71, 1.22)  1.07 (0.78, 1.47) | 86  NA | 0.787 |
| Geographic location  United States  Europe  Australia  Asia | 3  4  1  0 | 0.82 (0.62, 1.10)  1.11 (1.01, 1.22)  1.31 (0.97, 1.77)  - | 77  19  NA  - | 0.596  0.175  - |
| Duration of follow-up, y  <10  ≥10 | 4  4 | 1.24 (1.07, 1.45)  0.91 (0.74, 1.11) | 0  85 | 0.101 |
| Number of cases  <1000  ≥1000 | 4  4 | 1.24 (1.07, 1.45)  0.91 (0.74, 1.11) | 0  85 | 0.101 |
| Outcome assessment  Blood measurements  Self-report, validated Medical record | 3  4  1 | 0.97 (0.58, 1.65)  1.08 (0.91, 1.28)  1.05 (0.95, 1.17) | 89  64  NA | 0.787  0.707 |
| ROBINS-I  Low/moderate  Serious | 8  0 | 1.03 (0.89, 1.20)  - | 76  - | - |
| Adjustment for family history of diabetes  Yes  No | 4  4 | 0.98 (0.72, 1.34)  1.08 (0.91, 1.28) | 85  67 | 0.452 |

^1^ Summary RRs were calculated using a random effects model. Summary RR, Summary relative risk; 95% CI, 95% confidence interval, ^2^P_between_, P for heterogeneity between subgroups with meta-regression

Summary RRs and 95% CIs from linear dose-response meta-analysis between total omega-6 fatty acid intake per 1 g/d with T2D incidence by subgroups^1^

|  | Cohorts, n | Summary RR (95% CI) | I^2^ (%) | P_between_^2^ |
| --- | --- | --- | --- | --- |
| All studies | 8 | 0.99 (0.98, 1.00) | 70 |  |
| Sex  Women  Men | 5  1 | 1.00 (0.99, 1.01)  0.97 (0.95, 0.98) | 33%  NA | 0.043 |
| Geographic location  United States  Asia  Australia  Europe | 4  1  1  2 | 0.99 (0.97, 1.00)  0.99 (0.98, 1.00)  1.04 (1.00, 1.08)  1.00 (0.99, 1.01) | 68  NA  NA  0.0 | 0.112  0.166  0.077 |
| Duration of follow-up, y  <10  ≥10 | 3  5 | 1.00 (0.98, 1.02)  0.99 (0.98, 1.00) | 60  78 | 0.566 |
| Number of cases  <1000  ≥1000 | 1  7 | 1.04 (1.00, 1.08)  0.99 (0.98, 1.00) | NA  67 | 0.077 |
| Outcome assessment  Self-report, validated Medical record | 7  1 | 0.99 (0.98, 1.00)  1.01 (0.99, 1.02) | 68  NA | 0.363 |
| ROBINS-I  Low/moderate  Serious | 8  0 | 0.99 (0.98, 1.00)  - | 70  - | - |
| Adjustment for family history of diabetes  Yes  No | 5  3 | 0.99 (0.98 1.00)  1.00 (0.99, 1.02) | 67  67 | 0.223 |

^1^ Summary RRs were calculated using a random effects model. Summary RR, Summary relative risk; 95% CI, 95% confidence interval, ^2^P_between_, P for heterogeneity between subgroups with meta-regression

Summary RRs and 95% CIs from linear dose-response meta-analysis between linoleic acid intake per 1 g/d with T2D incidence by subgroups^1^

|  | Cohorts, n | Summary RR (95% CI) | I^2^ (%) | P_between_^2^ |
| --- | --- | --- | --- | --- |
| All studies | 6 | 0.99 (0.98, 1.01) | 65 |  |
| Sex  Women  Men | 3  1 | 1.00 (0.99, 1.00)  0.97 (0.96, 0.99) | 0  NA | 0.093 |
| Geographic location  United States  Europe | 3  3 | 1.01 (0.99, 1.02)  0.99 (0.97, 1.00) | 34  75 | 0.257 |
| Duration of follow-up, y  <10  ≥10 | 2  4 | 1.02 (0.99, 1.04)  0.99 (0.98, 1.00) | 0  65 | 0.146 |
| Number of cases  <1000  ≥1000 | 2  4 | 1.02 (0.99, 1.04)  0.99 (0.98, 1.00) | 0  65 | 0.146 |
| Outcome assessment  Self-report, validated Medical record | 5  1 | 0.99 (0.98, 1.00)  1.04 (0.99, 1.09) | 64  NA | 0.131 |
| ROBINS-I  Low/moderate  Serious | 6  0 | 0.99 (0.98, 1.01)  - | 65  - | - |
| Adjustment for family history of diabetes  Yes  No | 2  4 | 1.02 (0.99, 1.04)  0.99 (0.98, 1.00) | 0  65 | 0.146 |

^1^ Summary RRs were calculated using a random effects model. Summary RR, Summary relative risk; 95% CI, 95% confidence interval, ^2^P_between_, P for heterogeneity between subgroups with meta-regression

Summary RRs and 95% CIs from linear dose-response meta-analysis between total omega-3 fatty acid intake per 250 mg/d with T2D incidence by subgroups^1^

|  | Cohorts, n | Summary RR (95% CI) | I^2^ (%) | P_between_^2^ |
| --- | --- | --- | --- | --- |
| All studies | 5 | 1.03 (0.98, 1.08) | 87 |  |
| Sex  Women  Men | 3  0 | 1.07 (1.02, 1.12)  - | 67  - | - |
| Geographic location  United States  Asia  Australia  Europe | 1  1  1  2 | 1.02 (0.98, 1.07)  0.95 (0.91, 0.99)  1.09 (1.02, 1.16)  1.05 (0.96, 1.15) | NA  NA  NA  93 | 0.273  0.337  0.401 |
| Duration of follow-up, y  <10  ≥10 | 3  2 | 1.02 (0.94, 1.10)  1.05 (0.96, 1.15) | 87  93 | 0.812 |
| Number of cases  <1000  ≥1000 | 1  4 | 1.02 (0.97, 1.07)  1.09 (1.02, 1.16) | NA  88 | 0.260 |
| Outcome assessment  Self-report, validated Medical record | 4  1 | 1.04 (0.97, 1.11)  1.01 (0.99, 1.02) | 89  NA | 0.745 |
| ROBINS-I  Low/moderate  Serious | 5  0 | 1.03 (0.98, 1.08)  - | 87  - | - |
| Adjustment for family history of diabetes  Yes  No | 2  3 | 1.06 (0.99, 1.14)  1.01 (0.95, 1.07) | 81  87 | 0.527 |

^1^ Summary RRs were calculated using a random effects model. Summary RR, Summary relative risk; 95% CI, 95% confidence interval, ^2^P_between_, P for heterogeneity between subgroups with meta-regression

Summary RRs and 95% CIs from linear dose-response meta-analysis between long-chain omega-3 fatty acid intake per 250 mg/d with T2D incidence by subgroups^1^

|  | Cohorts, n | Summary RR (95% CI) | I^2^ (%) | P_between_^2^ |
| --- | --- | --- | --- | --- |
| All studies | 16 | 1.06 (1.00, 1.11) | 79 | - |
| Sex  Women  Men | 5  3 | 1.09 (0.99, 1.19)  1.01 (0.92, 1.11) | 76  44 | 0.300 |
| Geographic location  United States  Europe  Asia  Australia | 6  5  4  1 | 1.14 (1.07, 1.22)  1.01 (0.99, 1.04)  0.92 (0.85, 0.99)  1.07 (0.94, 1.22) | 70  4  0  NA | 0.113  0.021  0.000 |
| Duration of follow-up, y  <10  ≥10 | 8  8 | 0.99 (0.92, 1.06)  1.11 (1.03, 1.18) | 43  87 | 0.021 |
| Number of cases  <1000  ≥1000 | 8  8 | 1.02 (0.96, 1.08)  1.08 (1.01, 1.16) | 0  89 | 0.274 |
| Outcome assessment  Self-report, validated  Blood measurements  Medical record | 11  4  1 | 1.07 (1.01, 1.15)  0.99 (0.92, 1.08)  1.01 (0.99, 1.04) | 79  0  NA | 0.714  0.447 |
| ROBINS-I  Low/moderate  Serious | 14  2 | 1.07 (1.01, 1.13)  0.96 (0.87, 1.06) | 81  0 | 0.285 |
| Adjustment for family history of diabetes  Yes  No | 8  8 | 1.07 (0.98, 1.17)  1.03 (0.99, 1.08) | 82  42 | 0.754 |

^1^ Summary RRs were calculated using a random effects model. Summary RR, Summary relative risk; 95% CI, 95% confidence interval, ^2^P_between_, P for heterogeneity between subgroups with meta-regression

Summary RRs and 95% CIs from linear dose-response meta-analysis between alpha linolenic acid intake per 250 mg/d with T2D incidence by subgroups^1^

|  | Cohorts, n | Summary RR (95% CI) | I^2^ (%) | P_between_^2^ |
| --- | --- | --- | --- | --- |
| All studies | 11 | 1.01 (0.98, 1.05) | 66 | - |
| Sex  Women  Men | 3  2 | 1.05 (0.98, 1.14)  0.99 (0.92, 1.08) | 76  NA | 0.523 |
| Geographic location  United States  Asia  Europe  Australia | 3  2  5  1 | 0.98 (0.90, 1.07)  1.01 (0.84, 1.21)  1.00 (0.98, 1.02)  1.16 (1.07, 1.27) | 36  85  0  NA | 0.054  0.546  0.957 |
| Duration of follow-up, y  <10  ≥10 | 6  5 | 1.02 (0.95, 1.10)  1.00 (0.98, 1.03) | 79  17 | 0.947 |
| Number of cases  <1000  ≥1000 | 6  5 | 1.06 (1.00, 1.12)  0.98 (0.95, 1.02) | 48  55 | 0.076 |
| Outcome assessment  Self-report, validated  Blood measurements  Medical record | 7  3  1 | 1.01 (0.96, 1.06)  1.06 (0.94, 1.20)  0.99 (0.97, 1.01) | 75  34  NA | 0.629  0.792 |
| ROBINS-I  Low/moderate  Serious | 9  2 | 1.01 (0.98, 1.05)  0.79 (0.32, 1.98) | 71  46 | 0.468 |
| Adjustment for family history of diabetes  Yes  No | 5  6 | 1.02 (0.99, 1.05)  1.01 (0.96, 1.08) | 0  79 | 0.778 |

^1^ Summary RRs were calculated using a random effects model. Summary RR, Summary relative risk; 95% CI, 95% confidence interval, ^2^P_between_, P for heterogeneity between subgroups with meta-regression

Summary RRs and 95% CIs from linear dose-response meta-analysis between *trans*-fatty acid intake per 1 g/d with T2D incidence by subgroups^1^

|  | Cohorts, n | Summary RR (95% CI) | I^2^ (%) | P_between_^2^ |
| --- | --- | --- | --- | --- |
| All studies | 7 | 1.00 (0.95, 1.06) | 67 | - |
| Sex  Women  Men | 4  1 | 1.00 (0.92, 1.09)  0.96 (0.90, 1.03) | 79  NA | 0.572 |
| Geographic location  United States  Europe  Asia  Australia | 5  2  0  0 | 1.00 (0.94, 1.07)  1.00 (0.81, 1.24)  -  - | 78  0  -  - | 0.848  -  - |
| Duration of follow-up, y  <10  ≥10 | 2  5 | 0.98 (0.91, 1.06)  1.01 (0.94, 1.09) | 0  78 | 0.786 |
| Number of cases  <1000  ≥1000 | 2  5 | 1.08 (0.98, 1.20)  0.99 (0.93, 1.06) | 0  73 | 0.257 |
| Outcome assessment  Self-report, validated  Blood measurements | 4  3 | 0.95 (0.92, 0.99)  1.08 (1.03, 1.14) | 0  0 | 0.021 |
| ROBINS-I  Low/moderate  Serious | 7  0 | 1.00 (0.95, 1.06)  - | 67  - | - |
| Adjustment for family history of diabetes  Yes  No | 4  3 | 1.01 (0.94, 1.08)  1.00 (0.88, 1.14) | 63  67 | 0.864 |

^1^ Summary RRs were calculated using a random effects model. Summary RR, Summary relative risk; 95% CI, 95% confidence interval, ^2^P_between_, P for heterogeneity between subgroups with meta-regression
